# Development and validation of an environmental DNA assay to detect federally threatened groundwater salamanders in central Texas

**DOI:** 10.1371/journal.pone.0288282

**Published:** 2023-07-10

**Authors:** Zachary C. Adcock, Michelle E. Adcock, Michael R. J. Forstner

**Affiliations:** 1 Department of Biology, Texas State University, San Marcos, Texas, United States of America; 2 Cambrian Environmental, Austin, Texas, United States of America; Universitat Zurich, SWITZERLAND

## Abstract

The molecular detection of DNA fragments that are shed into the environment (eDNA) has become an increasingly applied tool used to inventory biological communities and to perform targeted species surveys. This method is particularly useful in habitats where it is difficult or not practical to visually detect or trap the target organisms. Central Texas *Eurycea* salamanders inhabit both surface and subterranean aquatic environments. Subterranean surveys are challenging or infeasible, and the detection of salamander eDNA in water samples is an appealing survey technique for these situations. Here, we develop and validate an eDNA assay using quantitative PCR for *E*. *chisholmensis*, *E*. *naufragia*, and *E*. *tonkawae*. These three species are federally threatened and constitute the *Septentriomolge* clade that occurs in the northern segment of the Edwards Aquifer. First, we validated the specificity of the assay *in silico* and with DNA extracted from tissue samples of both target *Septentriomolge* and non-target amphibians that overlap in distribution. Then, we evaluated the sensitivity of the assay in two controls, one with salamander-positive water and one at field sites known to be occupied by *Septentriomolge*. For the salamander-positive control, the estimated probability of eDNA occurrence (ψ) was 0.981 (SE = 0.019), and the estimated probability of detecting eDNA in a qPCR replicate (*p*) was 0.981 (SE = 0.011). For the field control, the estimated probability of eDNA occurring at a site (ψ) was 0.938 (95% CRI: 0.714–0.998). The estimated probability of collecting eDNA in a water sample (θ) was positively correlated with salamander relative density and ranged from 0.371 (95% CRI: 0.201–0.561) to 0.999 (95% CRI: 0.850– > 0.999) among sampled sites. Therefore, sites with low salamander density require more water samples for eDNA evaluation, and we determined that our site with the lowest estimated θ would require seven water samples for the cumulative collection probability to exceed 0.95. The estimated probability of detecting eDNA in a qPCR replicate (*p*) was 0.882 (95% CRI: 0.807–0.936), and our assay required two qPCR replicates for the cumulative detection probability to exceed 0.95. In complementary visual encounter surveys, the estimated probability of salamanders occurring at a known-occupied site was 0.905 (SE = 0.096), and the estimated probability of detecting salamanders in a visual encounter survey was 0.925 (SE = 0.052). We additionally discuss future research needed to refine this method and understand its limitations before practical application and incorporation into formal survey protocols for these taxa.

## Introduction

The Edwards-Trinity Aquifer system of central Texas provides habitat for approximately 15 species of endemic, plethodontid salamanders of the genus *Eurycea* [1]. These taxa are aquatic and inhabit springs and spring-fed creeks as well as subterranean water in alluvium and the pores, conduits, and caves of eroded karst limestone [1, 2]. All species likely utilize subterranean water to some degree, although the extent of use is not known for several taxa. Some are stygobiontic (obligate aquifer-dwellers), such as *E*. *rathbuni* and *E*. *waterlooensis* [3, 4], while others, at minimum, migrate to subsurface habitat for oviposition [5–9] or as a temporary refuge from drying surface conditions [2, 10, 11]. Epigean (surface) populations typically occur proximate to a spring outlet or a portion of the creek gaining subterranean water [2, 9, but also see 12, 13].

Central Texas *Eurycea* are of conservation concern because of their small distributions on a landscape undergoing rapid urbanization [1, 14]. The United States Fish and Wildlife Service (USFWS) protects seven species [15–19], Texas Parks and Wildlife Department (TPWD) protects four additional species [20], and more listing petitions are predicted [1]. Consequently, surveys are commonly conducted for these taxa to determine occupancy of a target locality [13], population size [8, 21], or species distribution [22]. Surface habitat and accessible cave streams can be surveyed by searching under and in submerged cover objects and with various trapping techniques [2, 23–25], but some of these locations require considerable effort to detect salamanders [13, 22]. Biological surveys in subterranean water are challenging [26–28], and subterranean salamander surveys are typically conducted by trapping spring outlets and wells when human access is not possible [22, 24, 29]. However, the molecular detection of DNA fragments that are shed into the environment (eDNA) potentially offers a powerful tool to survey both subterranean and surface water for central Texas *Eurycea*. The molecular detection of eDNA is especially useful in aquatic habitats where it is difficult or not practical to visually detect or trap the target taxa [27, 28, 30–32]. Of particular relevance, previous researchers demonstrate that eDNA surveys are effective for rare, aquatic amphibians in swift-flowing lotic systems [32–35], including *Urspelerpes brucei* and *E*. *bislineata* which are also headwater stream-dwelling plethodontids [36, 37]. Further, eDNA methods successfully detect stygobiontic salamanders in European karst aquifers [38, 39].

Here, we sought to develop and validate an eDNA assay using quantitative PCR (qPCR) for three species of central Texas *Eurycea*: *E*. *chisholmensis*, *E*. *naufragia*, and *E*. *tonkawae*. These three species constitute the *Septentriomolge* clade that occurs in the northern segment of the Edwards Aquifer [1, 4], and all three species are listed as federally threatened [18, 19]. The goals of this study were to 1) develop a cost-effective and easily implemented qPCR assay for *Septentriomolge*, 2) validate the specificity of the assay *in silico* and with DNA extracted from tissue samples of both target *Septentriomolge* and non-target amphibians that overlap in distribution, 3) evaluate the sensitivity of the assay using samples from *Septentriomolge-*positive water, 4) evaluate the sensitivity of the assay using water samples from *Septentriomolge-*occupied sites, and 5) explore the effects of salamander relative density and water conditions on the collection and detection of *Septentriomolge* eDNA.

## Materials and methods

### Quantitative PCR assay design

We developed a single assay to detect all three *Septentriomolge* species because they demonstrate minimal genetic divergence, approximately 98% similarity, in their published mitochondrial DNA (mtDNA) *cytochrome b* (*cytb*) gene [4, 40, 41]. The distributions of the three species are not known to overlap [1], which allows locality to aid in species identification. Further, the legal implications of survey results are the same because the state and federal listing designations of each species are identical [18–20].

We chose qPCR over traditional PCR because it is portrayed as precise, sensitive, and specific to target taxa [34, 42, 43]. We developed primers and a fluorescent probe specific to a segment of the *cytb* gene for our target taxa using the primer design tool in Geneious v.11 (https://www.geneious.com). Geneious’ primer design tool implements a modified version of Primer3 software [44, 45]. We obtained *cytb* sequences from GenBank and used all nine published haplotypes for *Septentriomolge* [41] and 88 sequences (S1 Table) from the 23 non-target amphibian species that overlap in distribution [46] to exclude non-target taxa by design. If *cytb* sequences were not available for a non-target, overlapping amphibian, then we used a congener’s sequence as a surrogate (S1 Table). We designed the primer and probe combination to have no base pair mismatches with *E*. *chisholmensis*, *E*. *naufragia*, and *E*. *tonkawae*, but at least five total base pair mismatches with all non-target amphibian sequences. Integrated DNA Technologies, Inc. (IDT, Coralville, IA, USA) provided the designed oligonucleotides, and the probe utilized a 5’ fluorescent dye (6–FAM), an internal ZEN quencher, and a 3’ Iowa Black FQ quencher (Table 1).

**Table 1:** Oligonucleotides used for the *Septentriomolge*-specific qPCR assay.

Oligonucleotide	Name	Sequence
Forward primer	SEPT.1-26-F	5’–TTCTAACAGGCCTATTTCTCGC–3’
Reverse primer	SEPT.1-110-R	5’–ACATCACGGCAAATGTGGG–3’
Probe	SEPT.1-67-P	5’–6–FAM–ACTACTTCC–ZEN–GCATTTTCCTCCGTGG–3’–IBFQ
Target fragment		5’–TTCTAACAGGCCTATTTCTCGCAATACACTATACTGCAGAYACTACTTCCGCATTTTCCTCCGTGGCCCACATTTGCCGTGATGT–3’

We tested the specificity of the assay *in silico* and with DNA extracted from tissues. We note that specificity is the probability of a true negative, and we consider the assay specific if the probability of a false positive is low (i.e., low probability of detecting *Septentriomolge* DNA when *Septentriomolge* DNA is absent). We tested specificity *in silico* using Primer-BLAST (https://www.ncbi.nlm.nih.gov/tools/primer-blast/) to identify any sequences from non-target taxa with less than five total base pair mismatches. We additionally tested specificity using DNA extracted from tissue of target *Septentriomolge* taxa and non-target amphibians that overlap the salamanders’ distribution. We used *Septentriomolge* tissue samples collected from six sites, including Stillhouse Hollow Spring (*E*. *tonkawae* type locality) and Cobbs Spring, which almost spans the latitudinal distribution of the clade [1]. We additionally used 43 tissue samples from non-target, overlapping amphibians that were collected from various projects in Texas. We tested at least two samples from each of the 12 overlapping genera [46], and in almost every case, we tested two samples from each overlapping non-target species (S2 Table).

### Site descriptions

**Fig 1:**
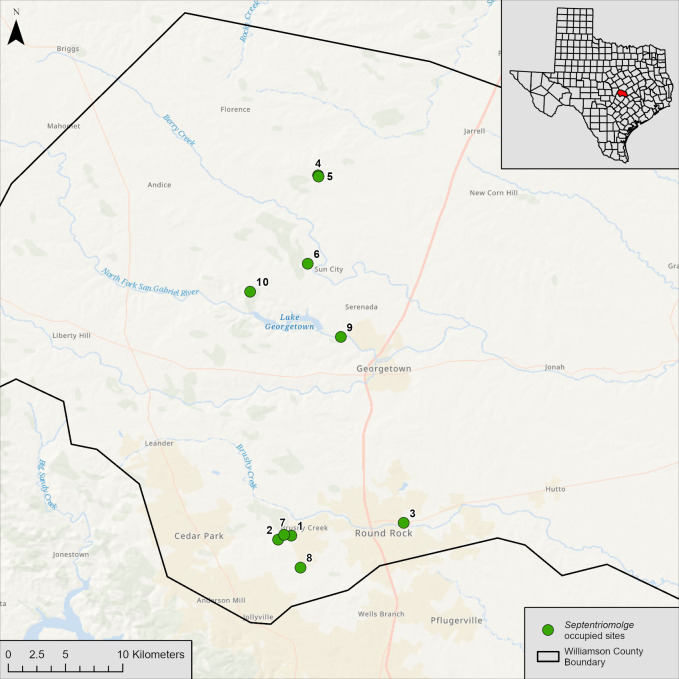
eDNA sampling locations in Williamson County, Texas, USA. Green circles denote field control sites that are known to be occupied by either *Eurycea chisholmensis*, *E*. *naufragia*, or *E*. *tonkawae* (*Septentriomolge* clade): 1) Avery Deer Spring, 2) Avery Springhouse Spring, 3) Brushy Creek Spring, 4) Cobbs Spring, 5) Cobbs Well, 6) Cowan Spring, 7) Hill Marsh Spring, 8) PC Spring, 9) Swinbank Spring, 10) Twin Springs. Spatial file sourced from the USGS National Map Viewer.

We collected water samples from salamander-positive controls and field control sites. Salamander-positive controls were collected from a known volume of spring water in which a salamander was held for a known amount of time, and field controls were collected from *Septentriomolge*-occupied sites (Fig 1). We used salamander-positive controls and field controls to test assay sensitivity. We note that sensitivity is the probability of a true positive, and we consider the assay sensitive if the probability of a false negative is low (i.e., low probability of not detecting *Septentriomolge* DNA when *Septentriomolge* DNA is present). *Septentriomolge*-occupied sites had regular visual encounter surveys, and displayed a range of relative abundances, including sites that averaged few (e.g., 0–2) to many (e.g., > 60) salamander detections per survey [9, 21, 47–49]. The distribution of salamanders at each field control site (i.e., the typical extent of the salamander population downstream of the spring outlet) was previously determined by visual encounter surveys [9, 21, 47–49].

### Water sample collection: Salamander-positive control

We collected salamander-positive control samples at Avery Deer, Avery Springhouse, Brushy Creek, Hill Marsh, and PC Springs (Fig 1) from May–July 2016. We captured and held salamanders in individual, sterile, plastic bags with 250 mL of spring water. We recorded the amount of time (minutes) the animals were held in the bag and measured the total length of salamanders with dial calipers to the nearest 0.1 mm. We collected 50 mL of water from each bag in sterile, single-use centrifuge tubes and vacuum filtered the 50 mL sample through a 47 mm diameter cellulose nitrate filter paper with a 0.45 μm pore size (Whatman^TM^, Cytiva, Malborough, MA, USA) in the field [32, 34, 35]. Filters were placed in 95% ethanol and stored at –20° C until DNA extraction. We collected a total of 53 salamander-positive samples among the five sites [S1 Appendix].

### Water sample collection: Field control

We collected field control water samples from December 2019 to January 2020 at 10 sites known to be occupied by *Septentriomolge* (Fig 1). Prior to sample collection, 1 L plastic water bottles were sterilized with 50% bleach, thoroughly rinsed with tap water, allowed to air dry, and sealed [34]. At sites with surface habitat (springs), we sampled at the spring outlet and at the typical downstream extent of the salamander population. We collected downstream samples before spring outlet samples. Prior to sample collection, we triple rinsed bottles in the field using water from the sampling location [34], and we collected three 1 L water samples at each sampling location [S2 Appendix]. We additionally collected one 1 L negative control of store-bought distilled water [34] per site. Water samples were stored on ice until filtration.

We also measured water temperature, pH, dissolved oxygen (DO), specific conductance (SC), and flow velocity at each sampling location. We measured water conditions with a Com-100 from HM Digital (Culver City, CA, USA), EcoTestr pH 2 from Oakton Instruments (Vernon Hills, IL, USA), HI 9147 from Hanna Instruments (Woonsocket, RI, USA), and a MF pro electromagnetic current meter from OTT Hydromet (Loveland, CO, USA). After sample collection, we performed a visual encounter survey for salamanders in accordance with USFWS survey protocol by searching in and under potential cover objects in the springs and with a modified leaf litter bag in the well [24]. We recorded the number of salamander detections and measured the survey area between the spring outlet and the downstream sampling location to estimate salamander relative density per site (Table 2). We note that visual encounter surveys did not account for imperfect detection, and we therefore report relative density [50]. We collected all samples and conducted all surveys in accordance with approved protocols from Texas State University’s Institutional Animal Care and Use Committee (IACUC 0417_0513_07), Texas Parks and Wildlife Department (Scientific Collecting Permits SPR-0102-191 and SPR-0319-056), and United States Fish and Wildlife Service (Federal Permits TE039544-1 and TE37416B-0).

**Table 2:** Visual encounter and eDNA survey results from field control sites in Williamson County, Texas, USA.

Site	*Eurycea* Taxon	Visual Encounter Surveys			eDNA Surveys	
		Survey Area (m^2^)	Salamander Detections	Salamander Relative Density	Positive Samples / Total Samples	Positive qPCRs / Total qPCRs
Avery Deer Spring	*E*. *tonkawae*	15.0	8	0.53	4/6	11/18
Avery Springhouse Spring	*E*. *tonkawae*	32.5	16	0.49	2/6	6/18
Brushy Creek Spring	*E*. *tonkawae*	25.0	0	0	2/6	6/18
Cobbs Spring	*E*. *chisholmensis*	28.25	29	1.03	6/6	17/18
Cobbs Well	*E*. *chisholmensis*	0.75	3	4.00	3/3	9/9
Cowan Spring	*E*. *chisholmensis*	10.0	8	0.80	2/6	2/18
Hill Marsh Spring	*E*. *tonkawae*	58.0	12	0.21	2/6	6/18
PC Spring	*E*. *tonkawae*	3.0	1	0.33	5/6	11/18
Swinbank Spring	*E*. *naufragia*	41.75	60	1.44	5/6	14/18
Twin Springs	*E*. *chisholmensis*	9.0	1	0.11	2/6	6/18

We vacuum filtered water samples within 12 hours of sample collection through a 47 mm diameter cellulose nitrate filter paper with a 0.45 μm pore size (Whatman^TM^). Filters were cut in half, placed in 95% ethanol, and stored at –20° C until DNA extraction. Half of the filter was used for DNA extraction and the other half archived in a –80° C freezer to allow future testing. We were careful to avoid cross-contamination during sample collection, filtering, and filter storage. We sterilized filter funnels in the same manner as collection bottles, and we bleached and rinsed work benches, filter flasks, and vacuum equipment between sampling occasions [34, 51]. We always filtered negative field control samples last to identify potential false positives caused by contamination from either field collection or filtering procedures. We manipulated filters in sterile, single-use petri dishes, and we sterilized scissors, forceps, and probes between samples by washing in 50% bleach, rinsing with deionized water, rinsing with 95% ethanol, and flaming the equipment [34, 51, 52].

### DNA extraction and purification

For the assay specificity test, we extracted DNA from tissue samples with a Qiagen DNeasy® Blood and Tissue Kit (Qiagen, Inc., Venlo, Netherlands) and used the standard tissue protocol. For water samples, we extracted DNA from filters also with a Qiagen DNeasy® Blood and Tissue Kit but with the following modified protocol. We cut the filter halves into small pieces and allowed them to dry overnight prior to lysis steps. We increased the volume of Buffer ATL (675 μL) and proteinase K (75 μL) to completely immerse the filter paper but maintain the same 9:1 ratio as the Qiagen protocol [53, 54]. We allowed lysis (i.e., agitation and incubation at 56° C) to occur for approximately 24 hours. Buffer AL and ethanol volumes were each increased to 750 μL to maintain the same 1:1:1 ratio of lysis solution, Buffer AL, and ethanol as the Qiagen protocol [53, 54]. Because reagent volumes were increased, we had to centrifuge 600 μL of the sample at a time to bind DNA to the spin column until the entire sample was filtered. We did not load the filter into the spin column [53, 54]. Wash steps followed the standard protocol. During the elution step, we warmed Buffer AE to 45° C and only used 100 μL to concentrate the final DNA product. We re-centrifuged the 100 μL of DNA product through the spin column to increase the amount of DNA eluted. We archived unused DNA from tissues and water samples in a –80° C freezer for future analyses.

We sterilized all equipment used during extractions (e.g., scissors, forceps, probe) with 50% bleach, then rinsed with deionized water, then rinsed with 95% ethanol, and flamed between samples [34, 51, 52]. We decontaminated lab benches and pipettes with 50% bleach before and after extractions [51].

### Quantitative PCR

Prior to conducting qPCRs, we optimized primer annealing temperatures using conventional PCR and evaluated the results with gel electrophoresis. We developed a six-point standard curve from 1:10 serial dilutions of a synthetic gene provided by IDT which contained our 85 base pair sequences of interest. The standard curve ranged from 8x10^6^–80 gene fragment copies/μL. All qPCR plates included triplicate reactions for samples, standards, and no template negative controls (i.e., reactions without DNA). For DNA extracted from tissue samples (assay specificity test), we diluted high yield product at 1:10 and low yield product at 1:5. We estimated DNA yield using gel electrophoresis by visualizing band brightness compared to a standard ladder. We did not dilute any eDNA samples.

Each qPCR reaction was 25.0 μL and included 2x TaqMan® Fast Advanced Master Mix (Applied Biosystems, Foster City, CA, USA), 0.5 μM of each primer, 0.25 μM of the probe, 0.009 μM bovine serum albumin, nuclease-free water, and 5.0 μL of sample DNA. We thermocycled reactions on a StepOnePlus^TM^ Real-Time PCR System (Applied Biosystems, Foster City, CA, USA) with initial denaturation at 95° C for three minutes and then 40 cycles with five seconds of denaturation at 95° C and 30 seconds of annealing and extension at 61.5° C.

We evaluated qPCR results by reviewing the amplification plots using the StepOnePlus^TM^ software. We only considered a qPCR reaction as positive (i.e., successful amplification of DNA) if its fluorescent signal demonstrated clear, exponential amplification without sudden spikes or dips in the normalized fluorescent signal (Rn) vs. cycle number plot and also demonstrated a plateau in the ΔRn vs. cycle number plot [55]. A threshold cycle (C_T_) of 34 corresponded to our standard of approximately 80 gene fragment copies/μL, and we used this value as a benchmark to evaluate successful amplification. We considered reactions with a C_T_ > 34 as negative because exponential amplification was not clearly identified. We also considered 18 qPCR reactions with a C_T_ ≤ 34 as negative because the curve shape was not exponential or exhibited spikes or dips, which may indicate fluorescence not associated with amplified product, such as background noise from degraded probes [55]. We considered each qPCR reaction as a result, and therefore, each sample had three qPCR results that formed its detection history.

### Assay sensitivity analysis: Salamander-positive control

Several studies have documented the advantage of using site occupancy models to analyze eDNA data because eDNA detection is imperfect [56–58]. To evaluate assay sensitivity in our salamander-positive control, we used a classic occupancy model [59, 60] implemented in R [61] with the package ‘unmarked’ [62]. We estimated the probability of eDNA occurring in a salamander-positive sample (ψ) and the probability of detecting eDNA in a qPCR replicate (*p*) in a model without covariates, ψ(.)*p*(.). Additional analysis notes, data, and code are provided in S1 Appendix.

### Assay sensitivity analyses: Field control

For our field control, we used multiscale occupancy models [63] to accommodate our three-level nested sampling design of multiple qPCR replicates per water sample and multiple water samples per site [56–58]. We fit models in R [61] with the package ‘ednaoccupancy’ which uses a Bayesian Markov Chain Monte Carlo (MCMC) algorithm [64]. Estimated parameters included the probability of *Septentriomolge* eDNA occurring at a site (ψ), the conditional probability of collecting *Septentriomolge* eDNA in a water sample given that eDNA was present at the site (θ), and the conditional probability of detecting eDNA in a qPCR replicate given that eDNA was present in the water sample (*p*).

We first determined that there was no difference in the probability of eDNA occurring at the spring outlet compared to downstream sampling locations, and we excluded this covariate from subsequent analyses [S2 Appendix]. Next, we constructed models with salamander relative density and water conditions as covariates. We considered the results of the concurrent visual encounter survey as a predictor for collecting eDNA in a water sample, as previous studies document a positive correlation between target species density and the probability of collecting eDNA in a sample [33–35, 56]. We also considered parameters that may influence the probability of collecting eDNA in a water sample due to DNA degradation or dilution, such as temperature, SC, DO, and flow velocity [28, 65–68]. We considered the effect of SC on the detection of eDNA in a qPCR replicate because of potential PCR inhibition [68, 69]. We excluded pH from the models because pH minimally deviated from neutral at all sampling locations. If multiple measurements of water conditions were recorded at a site, we used the average value as the covariate [S3 Appendix]. We did not consider any site covariates, as all sampled sites were known to be occupied. We scaled and centered all covariate data prior to fitting models [70]. We fit eight total models, including six combinations of salamander density and water condition covariates [S3 Appendix], a null model, and a full model, ψ(.)θ(density + temperature + SC + DO + flow velocity)*p*(SC). We ran 50,000 iterations of the MCMC algorithm for each of the multiscale occupancy models. We used a burn-in of 5,000 to discard the initial transient region of the chains, and we did not thin values from the chains.

We performed model selection using the Widely Applicable Information Criterion (WAIC; [71, 72]) and Posterior-Predictive Loss Criterion (PPLC; [73]) functions in ‘ednaoccupancy’ [64]. Both methods penalize models with more parameters, and models with the lowest WAIC and PPLC values are favored [64, 71–73]. We inspected Markov chain convergence and levels of autocorrelation for the top model [S3 Appendix]. We report the posterior medians with 95% credible intervals (95% CRIs) for the estimated parameters of the top model. We considered an estimated parameter affected by a covariate when the 95% CRI of the slope estimate did not include zero [56, 64, 70]. We acknowledge that our sample size is low (i.e., 10 sites), and we emphasize that our primary objective was to estimate site occupancy to evaluate assay sensitivity. The inclusion of covariates is intended to be exploratory to inform future studies.

We calculated two derived parameters from the top model to assess if the number of water samples and qPCR replicates were adequate. We used the estimated median probability of collecting eDNA in a water sample (θ) to calculate the cumulative collection probability (θ*) after *j* water samples, assuming *Septentriomolge* eDNA was present at the site, using the equation θ* = 1 –(1 – θ)^*j*^ [56, 57, 74]. We calculated θ* for four sites that represent the range of θ estimates. We then used the estimated median probability of detecting eDNA in a qPCR replicate (*p*) to calculate the cumulative detection probability (p*) after *k* qPCR replicates, assuming *Septentriomolge* eDNA was present in a water sample, using the equation p* = 1 –(1 –*p*)^*k*^ [56, 57, 74]. We determined the number of water samples and qPCR replicates necessary for each cumulative probability to exceed 0.95, which is a standard benchmark for surveys [24, 56, 57, 74]. Additional analyses notes, data, and code are provided in S3 Appendix.

We additionally fit a classic occupancy model [59, 60] in ‘unmarked’ [62] using visual encounter survey data to provide a comparison of site occupancy estimates between eDNA and standard visual encounter surveys. We included detection/nondetection data from visual encounter surveys conducted on the day of eDNA sample collection (Table 2) and surveys from the three previous months which corresponds to the early portion of the *Septentriomolge* breeding season [8, 9, 21]. We assumed demographic closure over this timeframe, and consequently, each site had 2–4 temporal survey replicates. We estimated the probability of salamanders occurring at a site (ψ) and the probability of detecting salamanders in a visual encounter survey (*p*) in a model without covariates, ψ(.)*p*(.). Additional analysis notes, data, and code are provided in S4 Appendix.

## Results

### Assay specificity

Our final *Septentriomolge*-specific assay targeted an 85 base pair fragment of *cytb* that had a single degenerate base among the nine documented haplotypes for the three species (Table 1). The Primer-BLAST did not identify any published sequences with less than five total base pair mismatches to the primers and probe from non-target taxa that overlap in distribution (i.e., no false positives). Further, DNA extracted from non-target, overlapping amphibians (*n* = 43) did not amplify in any of the qPCR replicates (*n* = 129; S2 Table), and none of the no template negative qPCR controls amplified. DNA extracted from target *Eurycea* (*n* = 6) amplified in every qPCR replicate (*n* = 18; S2 Table). Therefore, we report no false positives or false negatives from *in silico* or tissue sample trials.

### Assay sensitivity: Salamander-positive control

We report no false positives from negative field control samples nor from the no template negative qPCR controls. Captured salamanders ranged from 17.0–75.0 mm ($\bar{x}$ = 49.8, SD = 15.2 mm) total length, and the retention time in water ranged from 52–305 minutes ($\bar{x}$ = 139.2, SD = 59.0 minutes). However, we did not model the influence of these covariates on the probability of occurrence of eDNA because we only had one false negative in this control. We detected *Septentriomolge* eDNA in 52 of 53 salamander-positive samples and 153 of 159 qPCR replicates. The estimated probability of eDNA occurring in a salamander-positive sample was 0.981 (SE = 0.019), and the estimated probability of detecting eDNA in a qPCR replicate was also 0.981 (SE = 0.011).

### Assay sensitivity: Field control

We detected *Septentriomolge* eDNA at all ten field control sites, and therefore, we report no false negatives (Table 2). We again did not detect eDNA in any negative field control samples nor any of the no template negative qPCR controls (i.e., no false positives). Cumulatively, we detected eDNA in 33 of 57 water samples and 88 of 171 qPCR replicates (Table 2). Among the ten sites, salamander relative density ranged from 0.0–4.0 detections/m^2^ ($\bar{x}$ = 0.89, SD = 1.18 detections/m^2^), water temperature ranged from 11.8–18.6°C ($\bar{x}$ = 15.5, SD = 2.01°C), DO ranged from 2.8–9.1 mg/L ($\bar{x}$ = 6.2, SD = 1.60 mg/L), SC ranged from 609–907 μS/cm ($\bar{x}$ = 755.7, SD = 100.3 μS/cm), and flow velocity ranged from 0.002–0.327 m/s ($\bar{x}$ = 0.085, SD = 0.093 m/s).

**Fig 2:**
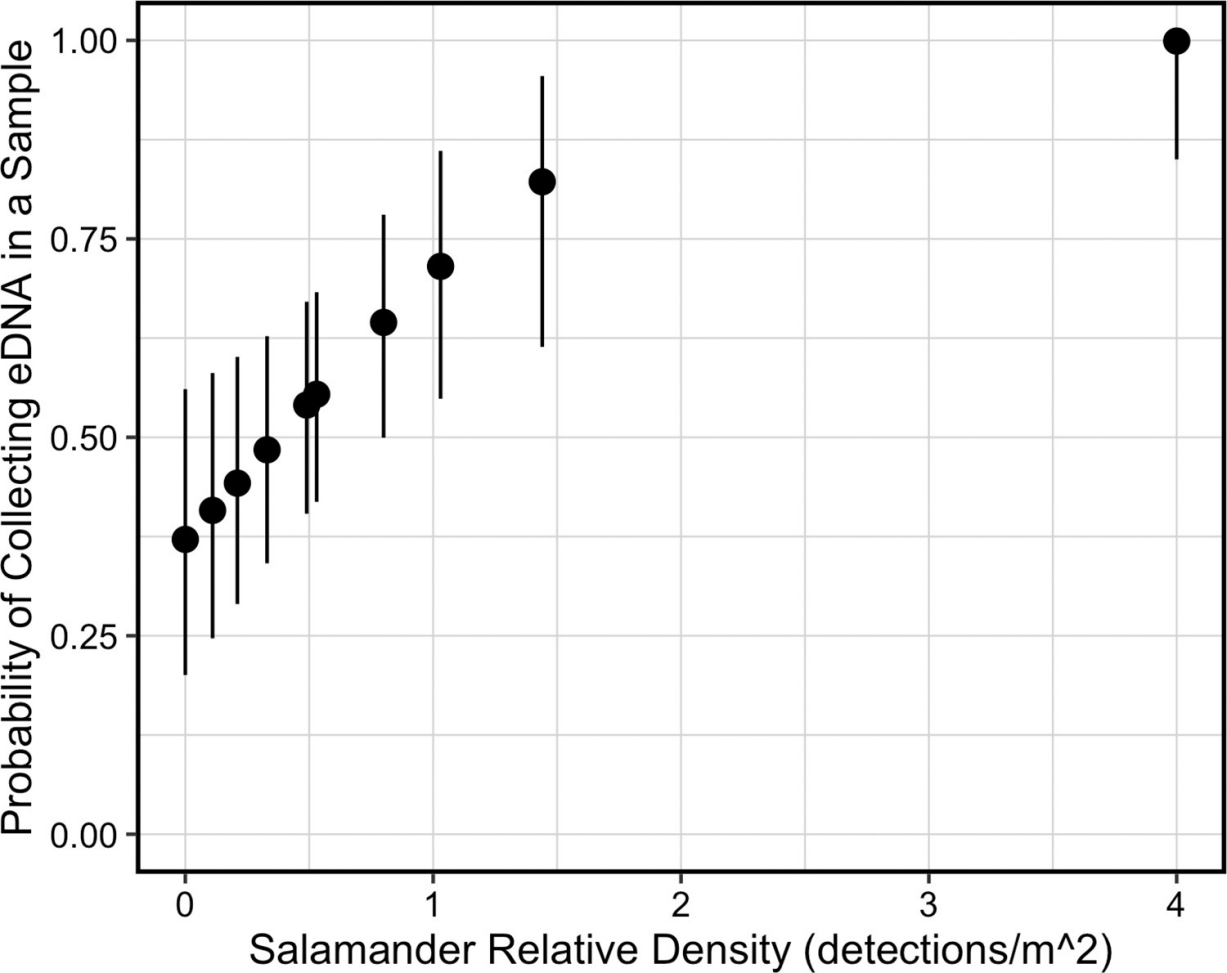
Estimated probability of collecting *Septentriomolge* eDNA in a water sample (θ) compared to *Septentriomolge* relative density at each site. We estimated probabilities from the top model, ψ(.)θ(density)*p*(.). Salamander relative density equals the number of salamander detections from visual encounter surveys divided by the survey area (m^2^) at each field control site. Symbols represent posterior medians with 95% credible intervals. We collected eDNA samples immediately before conducting visual encounter surveys.

Of the eight models we compared, the model with the lowest WAIC and PPLC included constant probability of eDNA occurrence at a site, eDNA collection probability influenced by salamander density, and constant detection probability of eDNA in a qPCR replicate (Table 3). For this top model, the estimated posterior median probability of eDNA occurring at a field control site (ψ) was 0.938 (95% CRI: 0.714–0.998). The estimated posterior median probability of collecting eDNA in a water sample (θ) ranged from 0.371 (95% CRI: 0.201–0.561) to 0.999 (95% CRI: 0.850– > 0.999). The probability of collecting eDNA in a water sample (θ) increased with salamander relative density as indicated by a 95% CRI of the slope estimate above zero (α_θ_: 0.306–1.896; Table 4, Fig 2). The estimated posterior median probability of detecting eDNA in a qPCR replicate (*p*) was 0.882 (95% CRI: 0.807–0.936).

**Table 3:** Model comparison using the Widely Applicable Information Criterion (WAIC) and Posterior-Predictive Loss Criterion (PPLC). Models are listed in order of increasing WAIC score. Criterion scores and predictive variance are presented for each method.

Model	WAIC		PPLC	
	Criterion	Predictive Variance	Criterion	Predictive Variance
ψ(.)θ(density)*p*(.)	30.62	3.93	26.56	11.20
ψ(.)θ(.)*p*(.)	30.80	4.10	26.62	11.26
ψ(.)θ(density + temperature + DO + SC + flow velocity)*p*(.)	31.61	4.89	26.73	11.39
ψ(.)θ(density)*p*(SC)	32.27	6.63	27.03	12.00
ψ(.)θ(temperature + DO + SC + flow velocity)*p*(.)	32.50	5.73	27.02	11.66
ψ(.)θ(.)*p*(SC)	33.24	7.53	27.53	12.47
ψ(.)θ(density + temperature + DO + SC + flow velocity)*p*(SC)	33.27	7.57	27.45	12.33
ψ(.)θ(temperature + DO + SC + flow velocity)*p*(SC)	34.58	8.78	28.05	12.78

**Table 4:** Summary of the Bayesian estimates of model parameters and Monte Carlo standard errors of Bayesian estimates (i.e., posterior mean, median, and 95% credible intervals) for the top multiscale occupancy model. The top model included salamander density as a predictor for the probability of collecting eDNA in a water sample, ψ(.)θ(density)*p*(.).

Parameter	Mean	Median	2.5%	97.5%
Bayesian estimates of model parameters				
β_ψ_ (intercept)	1.583	1.542	0.566	2.829
α_θ_ (intercept)	0.458	0.452	0.056	0.894
α_θ_ (density)	1.040	1.014	0.306	1.896
δ_*p*_ (intercept)	1.188	1.185	0.869	1.520
Monte Carlo standard errors of Bayesian estimates				
β_ψ_ (intercept)	0.0027	0.0034	0.0052	0.0099
α_θ_ (intercept)	0.0014	0.0018	0.0030	0.0034
α_θ_ (density)	0.0028	0.0036	0.0050	0.0062
δ_*p*_ (intercept)	0.0008	0.0010	0.0020	0.0022

**Fig 3:**
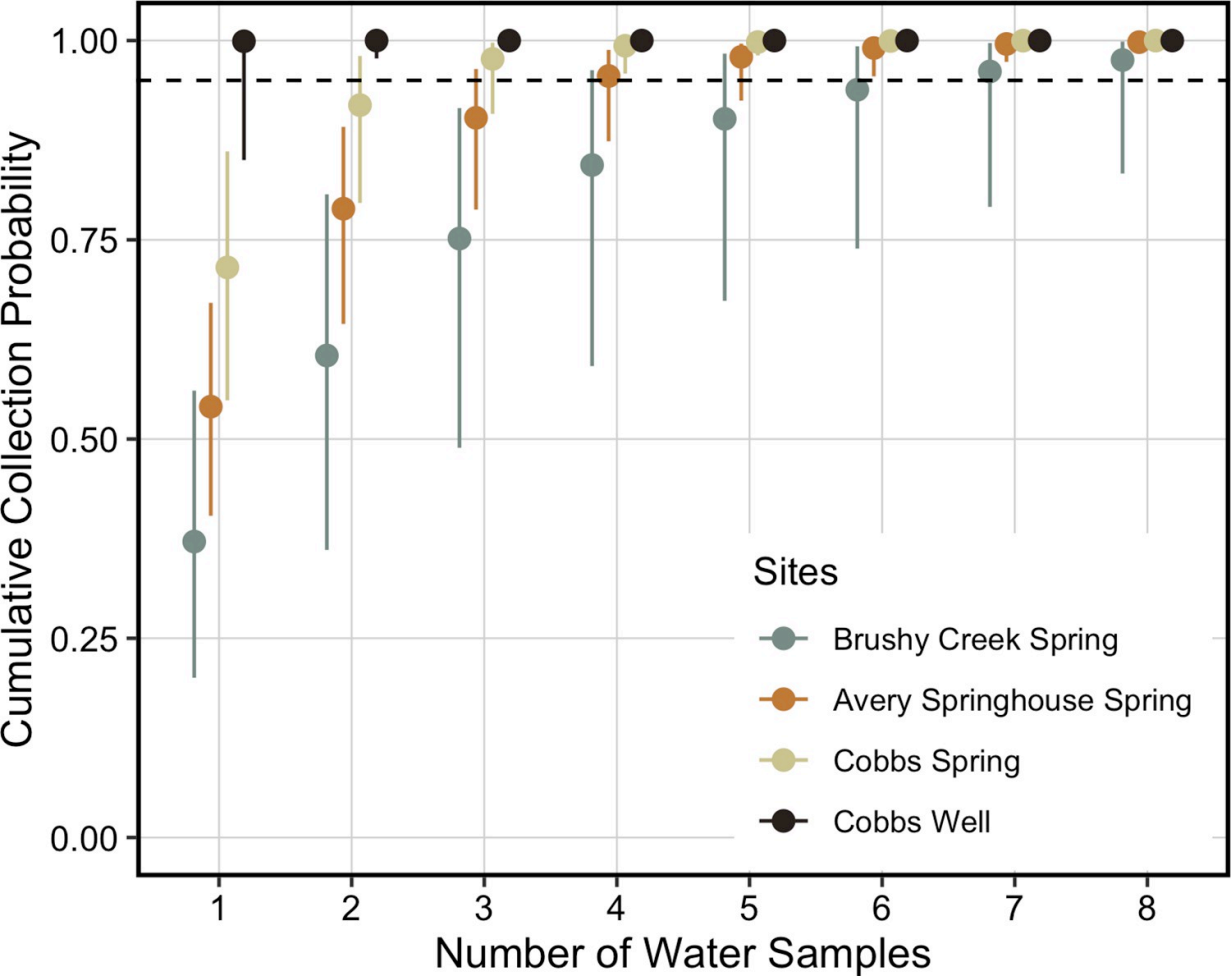
Cumulative probabilities of collecting *Septentriomolge* eDNA (θ*) after *j* water samples at four field control sites. Symbols represent posterior medians with 95% credible intervals. We calculated cumulative probabilities based on the median probabilities estimated from the top model, ψ(.)θ(density)*p*(.). We present θ* estimates for Brushy Creek Spring (θ = 0.371; 95% CRI: 0.201–0.561), Avery Springhouse Spring (θ = 0.541; 95% CRI: 0.404–0.671), Cobbs Spring (θ = 0.715; 95% CRI: 0.549–0.861), and Cobbs Well (θ = 0.999; 95% CRI: 0.850– > 0.999). The estimate of θ* assumes that *Septentriomolge* eDNA is present at a site [56, 74]. The dashed line denotes a cumulative probability of 0.95.

**Fig 4:**
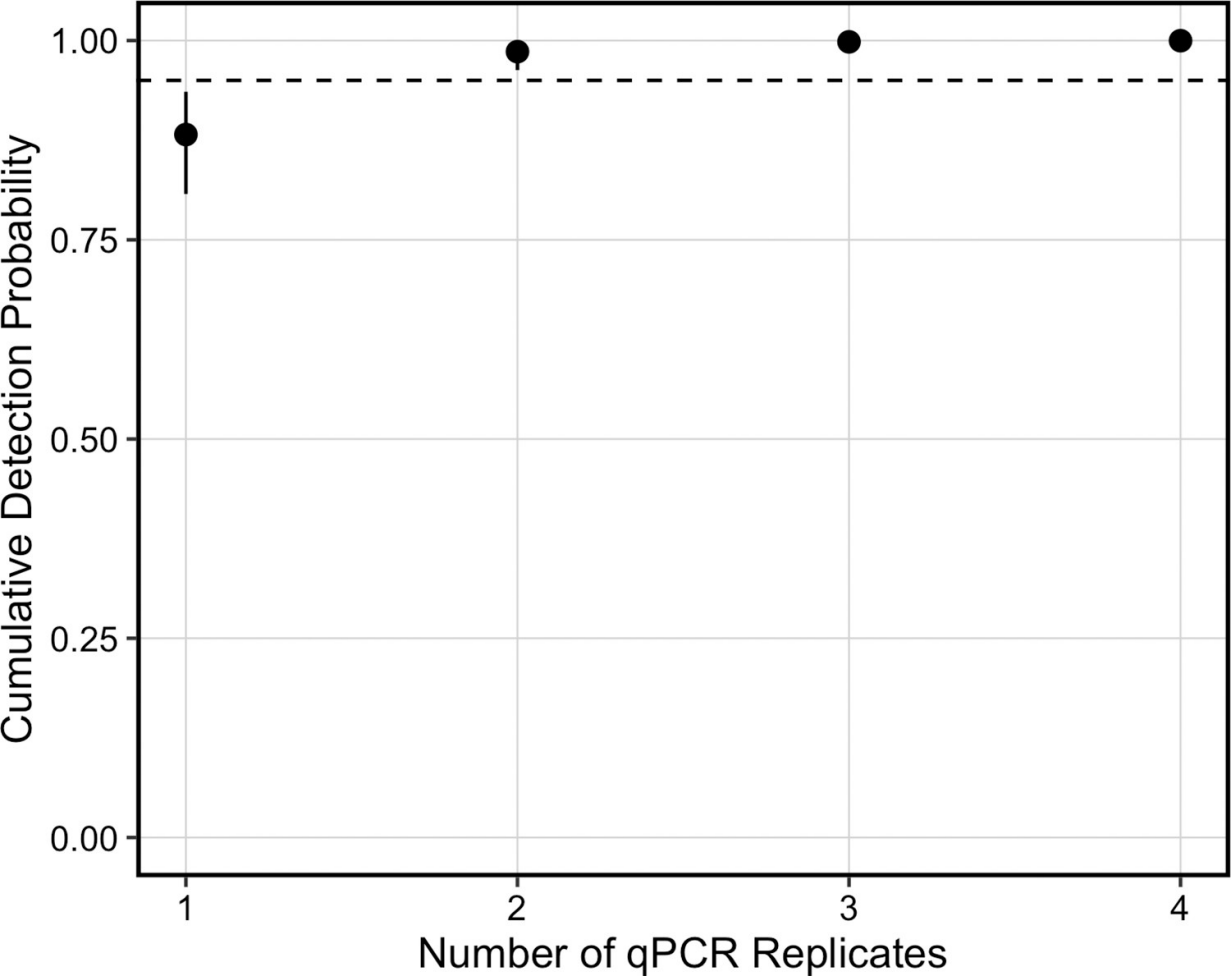
Cumulative probability of detecting *Septentriomolge* eDNA (p^*^) in *k* qPCR replicates at field control sites. Symbols represent posterior medians with 95% credible intervals. We calculated cumulative probabilities based on the median probability estimated from the top model, ψ(.)θ(density)*p*(.). The estimate of p^*^ assumes eDNA is present in a water sample [56, 74]. The dashed line denotes a cumulative probability of 0.95.

According to the estimates from the McArdle equation [74], the cumulative probability of collecting eDNA in at least one of the six water samples per site (θ*) ranged from 0.938 (95% CRI: 0.739–0.993) to > 0.999 (95% CRI: > 0.999). Seven water samples were required to obtain a θ* > 0.95 at our field control site with the lowest estimated θ, but only one water sample was necessary to exceed this metric at our field control site with the highest estimated θ (Fig 3). The cumulative probability of detecting eDNA in at least one of the three qPCR replicates (p*) was 0.998 (95% CRI: 0.993–0.999). Our assay required two qPCR replicates for p* to exceed 0.95 (Fig 4).

We detected *Septentriomolge* in 26 of 30 visual encounter surveys among the 10 known-occupied sites. This included visually detecting salamanders in every survey at eight sites, two of four surveys at Twin Springs, and zero of two surveys at Brushy Creek Spring [S4 Appendix]. The estimated probability of salamanders occurring at a site was 0.905 (SE = 0.096), and the estimated probability of detecting salamanders in a visual encounter survey was 0.925 (SE = 0.052).

## Discussion

### Accessibility of methods

Environmental DNA studies have utilized a variety of sample collection, preservation, DNA extraction, PCR, bioinformatic, and data analysis techniques [51, 75]. Our intention was to develop and test a methodology that uses affordable techniques that are accessible to most academic, government, and private researchers. Therefore, we collected and filtered water samples using reusable bottles and funnels with a strict decontamination procedure as opposed to more costly single-use materials. Further, water sample collection with off-site filtering allows field work to be conducted by individuals that may lack filtering and preservation equipment and expertise. Hinlo et al. [75] determined that filtering water samples through a cellulose nitrate filter, storing in ethanol or at ≤ 20° C, and extracting DNA with a Qiagen DNeasy kit was the most efficient procedure for both cost and DNA recovery. This collection, extraction, and storage procedure also uses readily available materials and techniques common to many laboratories.

### Assay specificity

Our qPCR eDNA assay is specific to *E*. *chisholmensis*, *E*. *naufragia*, and *E*. *tonkawae* within their distribution as demonstrated by the lack of amplification of DNA extracted from overlapping, non-target taxa. We did not identify any false positives from negative field controls or the no template negative qPCR controls which suggests that our reported field, lab, and decontamination procedures are sufficient for practical application. Because we report no false positives, a positive result indicates a high probability that *Septentriomolge* DNA was present.

### Assay sensitivity: Salamander-positive control

We had a high rate of success detecting *Septentriomolge* eDNA in salamander-positive control samples despite our small water sample volume (50 mL). Detection and occurrence probabilities were high enough that we did not need to evaluate the influence of salamander size and salamander retention time covariates. We note that we were required to retain salamanders in spring water from the collection site per USFWS protocol [24]. Our overwhelmingly positive results suggest the influence of potential background exogenous *Eurycea* eDNA was negligible, and most importantly, it does not diminish the primary purpose of the experiment, which was to demonstrate that our assay can detect *Septentriomolge* eDNA shed into the water.

### Assay sensitivity: Field control

The estimated probability of salamander occurrence at known-occupied sites was slightly higher using eDNA surveys compared to visual encounter surveys, but we note overlapping 95% CRIs. We detected *Septentriomolge* eDNA at all field control sites including one site, Brushy Creek Spring, where we did not detect salamanders during the complementary visual encounter surveys (Table 2). The surface habitat is highly modified at Brushy Creek Spring and visual encounter surveys without salamander detections are common [48]. Two samples from the spring outlet were positive for eDNA, indicating that we detected subterranean salamanders that were not available for detection by traditional visual encounter surveys.

We emphasize that the evaluation of covariates was intended to be exploratory to inform future work. We are careful to not overinterpret these results, as Schmidt et al. [56] recommended a sample size of at least 20 for site occupancy models. Salamander relative density influenced model parameters more than water conditions (Table 3). The probability of collecting eDNA in a water sample was positively correlated with salamander relative density at a site (Table 4, Fig 2), and this result is congruent with the general expectation that the probability of detecting a taxon increases with increasing density of the taxon [76]. Several studies have documented a positive correlation between target species density and eDNA detection [33–35, 56], but Plante et al. [37] found no relationship (*n* = 2) or a weak relationship (*n* = 1) between density and eDNA detection in three other plethodontid salamanders. We did not observe large differences in water condition variables among our sampled sites. Additional research is warranted on these covariates as previous studies determined that increasing temperature, DO, and salinity all increased DNA degradation, which decreased the probability of collecting eDNA in a water sample [65–68]. Increasing salinity also lowered qPCR detection in other studies due to PCR inhibition [68, 69]. We did not specifically measure calcium ion concentration or tannic acid, but both are known to inhibit PCR [77]. We collected samples in the winter when these sites have considerable leaf litter accumulation, and when we expect tannic acid and other PCR inhibitors from plant matter to be at their highest [78].

The probability of collecting eDNA in a sample, even when eDNA is present at a site, is < 1, and it is doubtful that the target eDNA will be collected in all samples [57, 58]. We report a large range in the conditional collection probability of eDNA in a water sample among our field control sites. As a result, the number of water samples required to obtain a θ* > 0.95 varied from one to seven 1 L samples. This result is important because we expect eDNA surveys to be applied at sites where visual detection of salamanders is difficult, which in some cases will be a result of low salamander density. Therefore, many water samples may be necessary to determine the true occupancy state of a site. For example, even at sites with frequent visual encounter detections (i.e., Avery Springhouse Spring and Cobbs Spring), three to four 1 L water samples may be necessary to obtain a θ* > 0.95 (Fig 3). Our qPCR eDNA assay had a high probability of detecting eDNA if present, requiring only two qPCR replicates for a cumulative probability of detection > 0.95 (Fig 4). These results are congruent with other studies that also separately modeled the water sampling and qPCR processes [56–58].

### Research needs

Further research is needed before we can make appropriate inferences from eDNA surveys for these taxa and this habitat. As already discussed, we encourage seasonal sampling and further testing the effects of water conditions. In addition, some sampling locations will benefit from suspended particulate removal prior to eDNA filtering [31, 79, S5 Appendix]. Allochthonous eDNA, which is eDNA deposited in the sampling area from outside sources [51, 80], warrants attention, as does molecular competition of non-target eDNA from abundant co-occurring taxa [43, 64, 81]. However, we consider eDNA dilution and transport to be priority research topics for this particular group of salamanders and habitat.

Our results indicate that the dilution of eDNA to undetectable levels may have influenced results at one sampling location. Cowan Spring had the fewest eDNA detections of any field control site even though it harbored the fourth highest salamander relative density (Table 2). We did not quantify spring discharge, but this site appears to have one of the largest discharges among the sampled springs. In hindsight, discharge volume would have been more informative than flow velocity because it provides information on the potential dilution of eDNA, and this covariate should be incorporated into future work [28, 78, 82]. We note that Wilcox et al. [43] successfully detected eDNA in qPCR reactions with only two copies of the target mtDNA fragment, and our threshold for positive results corresponded to approximately 80 gene fragment copies/μL. Our assay may need increased sensitivity to reduce false negatives when eDNA is diluted (e.g., low salamander density or large discharge systems). However, some techniques to increase assay sensitivity may also increase the likelihood of false positives [43, 83]. Many studies use > 40 PCR cycles to amplify low-copy samples, but extended cycles can cause false positives from non-specific primer or probe binding, fluorescent signal interference or wavelength interaction from other wells, and probe degradation at the end of runs [43, 55, 83–85]. Because *Septentriomolge* are federally listed, both false negative and false positive results have serious ecological, conservation, economic, and legal implications [51]. We did not identify any false positives in this study, so we did not estimate this parameter in our models. Recent studies suggest establishing detection thresholds to help correct false positive results [84, 85], and future applications that identify false positives should utilize models that allow for inference when both false negative and false positive errors occur [83, 86–89].

The amount of eDNA in a lotic system depends on the balance of eDNA shed into the water and the degradation and downstream transport of that eDNA [31, 66, 78]. Previous research concluded that eDNA persists for 8–25 days in aquaria and for 17 days in experimental ponds after removing the eDNA source [35, 90], but eDNA is flushed out of lotic systems (i.e., transported downstream and out of the study area) within 1–24 hours [35, 78]. In experimental translocation studies, salamander eDNA was detected 5 m but not detected 50 m downstream of the source [35], but fish translocation studies regularly detected eDNA hundreds of meters downstream of the source [78, 91]. Amphibian and fish eDNA has been detected > 20 km and > 100 km, respectively, from the source in wild populations [92, 93]. Understanding eDNA persistence and potential transport is critical for making inferences about occurrence and distribution of taxa because eDNA may be transported downstream of a target source and into unoccupied areas [27, 31, 82, 94]. No studies have investigated eDNA persistence or transport in groundwater habitats [27]. Ultraviolet light (UV) exposure, fluctuating temperatures, and microbial activity are important causes of eDNA degradation in surface water [43, 66]. Degradation rates are likely slower in subterranean water because it generally lacks UV exposure, has relatively constant and cooler water temperatures, and has reduced microbial activity [27, 38]. The degradation rate and transport potential of eDNA in groundwater habitats needs careful and substantial attention as this has the potential to misrepresent the occupancy state of a sampling location and the geographic distribution of species [27]. The entire *Septentriomolge* clade has an approximately 65 km latitudinal and 35 km longitudinal distribution [1, 18, 19]. Most central Texas *Eurycea* species have similarly small geographic distributions [1], and it is possible that eDNA could be transported a distance greater than the entire range of one of these species. Understanding potential eDNA transport in groundwater settings is further complicated because it requires detailed knowledge of groundwater flow paths [28].

Many eDNA research programs aim to estimate species density and abundance. Current research demonstrates mixed results, with some researchers documenting a positive correlation between eDNA quantity and target taxon abundance [33, 34, 42] and others reporting no relationship [37, 51, 95, 96]. Chambert et al. [97] developed a model to directly estimate animal density and associated uncertainty from eDNA data, but also reported important topics where the methodology needs further improvement. We recommend that future research focus on resolving the issues we highlighted herein to better understand inferences related to site occupancy before researchers attempt the difficult task of estimating density and abundance of central Texas *Eurycea* from eDNA.

### Future applications

Permitting regulations for *Septentriomolge* require documenting presence or inferring absence of the focal species at a location [24]. This “presence/absence” approach makes surveys for eDNA an attractive option, especially in situations where appropriate surface habitat may be limited or when subterranean trapping is not practical [27]. Under the current federal protocol, 15 visual encounter surveys plus seven 24-hour periods of bottle trapping or two weeks of drift net trapping are required to infer that *Septentriomolge* are absent in the surface and subsurface habitat at a location [24]. When subterranean surveys are not feasible, bottle and drift net traps are placed in the surface habitat and at the spring outlet to capture salamanders that may emerge from the subterranean environment [24]. Similar to other studies, our results indicate that traditional visual encounter surveys and surveys for eDNA have comparable efficiency in typical surface habitat [51]. However, there are likely situations where eDNA surveys will be more efficient than conducting the full suite of visual encounter and trapping surveys, even though eDNA surveys require expertise, costly laboratory supplies, and multiple actions (e.g., sample collection, sample filtering, DNA extractions, and qPCR). Surveys for eDNA are likely a better option than bottle traps and drift nets to determine the occupancy state of subterranean habitat because salamanders that do not exit the subterranean environment may still be detected [S2 Appendix]. Further, eDNA surveys cause less disturbance to salamanders and the habitat structure compared to visual encounter surveys which often requires displacing cover objects and capturing the animals [30, 37].

Surveys for *Septentriomolge* eDNA have the potential for important practical applications. Our assay can be applied to survey sites within the entire northern segment of the Edwards Aquifer to identify *Septentriomolge* populations for site occupancy and geographic distribution studies. The location of the sampling site would determine species identity according to Devitt et al. [1], or positive eDNA results could prompt additional surveys to obtain vouchers for species identification. The legal ramifications of a positive survey result (e.g., federal permitting requirements) are identical regardless of species designation because all three species currently share the same listing status [18–20]. However, we recommend supplementary research to refine this method before incorporation into formal survey protocols, especially regarding appropriate mechanisms to increase qPCR assay sensitivity without introducing false positives, in addition to research seeking to determine eDNA transport in aquifer environments. Until these topics are investigated, and the limitations of this technique are understood, this assay is best applied for research studies or as a complement to visual encounter and trapping surveys and not for regulatory application.

## Supporting information

S1 Appendix Additional notes, data, R code, and results for the salamander-positive control to evaluate the sensitivity of the *Septentriomolge* eDNA assay. (DOCX)

S2 Appendix A comparison of psi (ψ) and theta (θ) estimates from spring outlet and downstream sampling locations to inform the assay sensitivity, field control analysis. (DOCX)

S3 Appendix Additional notes, data, R code, and results for the field control to evaluate the sensitivity of the *Septentriomolge* eDNA assay. (DOCX)

S4 Appendix Additional notes, data, R code, and results for *Septentriomolge* visual encounter surveys to compare to the *Septentriomolge* eDNA assay. (DOCX)

S5 Appendix Application of the *Septentriomolge* eDNA assay at field sites with rare salamander detections and second order creeks downstream of occupied sites. (DOCX)

S1 Table Classification and GenBank accession number for target *Septentriomolge* and non-target amphibian mitochondrial DNA *cytochrome b* sequences used to design the *Septentriomolge*-specific qPCR assay. Non-target amphibians have a distributional overlap with *Septentriomolge* in Travis, Williamson, or Bell counties, Texas, USA [Dixon 2013]. *Ambystoma tigrinum* is not currently known to overlap in distribution but has potential for introduction because it is commonly used as fish bait [Dixon 2013]. *Eleutherodactylus coqui*, *Eleutherodactylus planirostris*, *Pseudacris feriarum*, and *Scaphiopus hurterii* are not known to overlap in distribution, but these sequences were used as surrogates or to supplement data for the overlapping genus.
(DOCX)

S2 Table Classification, collector number, collection county (Texas, USA), and results for target *Septentriomolge* and non-target amphibian tissue samples used to test the specificity of the *Septentriomolge* qPCR assay. Non-target amphibians have a distributional overlap with *Septentriomolge* in Travis, Williamson, or Bell counties, Texas, USA [Dixon 2013]. *Ambystoma tigrinum* is not currently known to overlap in distribution but has potential for introduction because it is commonly used as fish bait [Dixon 2013].
(DOCX)
