# Supplementary material for: Development and validation of an environmental DNA assay to detect federally threatened groundwater salamanders in central Texas
Source: PLoS One. 2023 Jul 10;18(7):e0288282. doi: 10.1371/journal.pone.0288282 (PMC10332605; doi:10.1371/journal.pone.0288282)
Supplement: S1 Appendix — (DOCX) [file pone.0288282.s001.docx]

**S1 Appendix. Additional notes, data, R code, and results for the salamander-positive control to evaluate the sensitivity of the *Septentriomolge* eDNA assay.**

Development and validation of an environmental DNA assay to detect federally threatened groundwater salamanders in central Texas

Zachary C. Adcock, Michelle E. Adcock, Michael R.J. Forstner

Table of Contents:

Additional Notes 2

Data 3

R code and Results 5

**Additional Notes**

We estimated the probability of eDNA occurring in a sample (ψ) and the probability of detecting eDNA in a qPCR replicate (*p*) for salamander-positive water samples. This represents the salamander-positive control to evaluate the sensitivity of the *Septentriomolge* eDNA assay.

- Sample = 50 mL of salamander-positive water
  - We collected samples from salamanders captured at Avery Deer Spring, Avery Springhouse Spring, Brushy Creek Spring, Hill Marsh Spring, and PC Spring.
  - We captured and held salamanders in individual, sterile, plastic bags with 250 mL of spring water.
  - We recorded the amount of time (minutes) the animals were held in the bag.
  - We measured the total length of salamanders with dial calipers to the nearest 0.1 mm.
  - We collected 50 mL of water from each bag in sterile, single-use centrifuge tubes.
  - We detected eDNA in 52 of 53 water samples.
  - We did not model the influence of total length or time covariates on the probability of occurrence of eDNA because we only had one false negative in the control.
- Replicate = qPCR result
  - We conducted three qPCR replicates per water sample. Therefore, each water sample had three qPCR results to form its detection history.
  - We detected eDNA in 153 of 159 qPCRs.
- Model
  - Null = psi(.) p(.)

**Data**

Table A. Covariates and detection histories for each salamander-positive water sample. We include collection site, sample identification number, salamander total length (mm), amount of time (minutes) the salamander was held in 250 mL of spring water, and qPCR results (‘0’ designates no amplification and ‘1’ designates amplification). (S1data)

| site | sample | tl | time | qPCR1 | qPCR2 | qPCR3 |
| --- | --- | --- | --- | --- | --- | --- |
| Avery Springhouse | 1013 | 62.0 | 115 | 1 | 1 | 1 |
| Avery Springhouse | 1014 | 38.7 | 107 | 1 | 1 | 1 |
| Avery Springhouse | 1015 | 39.2 | 115 | 1 | 1 | 1 |
| Avery Springhouse | 1016 | 64.6 | 118 | 1 | 1 | 1 |
| Avery Springhouse | 1017 | 63.3 | 115 | 1 | 1 | 1 |
| Avery Springhouse | 1018 | 64.4 | 127 | 1 | 1 | 1 |
| Avery Springhouse | 1019 | 55.7 | 106 | 1 | 1 | 1 |
| Avery Springhouse | 1020 | 60.3 | 114 | 1 | 1 | 1 |
| Avery Springhouse | 1021 | 59.9 | 121 | 1 | 1 | 1 |
| Avery Springhouse | 1022 | 34.0 | 125 | 1 | 1 | 1 |
| Avery Springhouse | 1023 | 31.1 | 140 | 1 | 1 | 1 |
| Avery Springhouse | 1024 | 26.2 | 152 | 1 | 1 | 1 |
| Avery Springhouse | 1025 | 23.5 | 160 | 1 | 1 | 1 |
| Avery Deer | 1026 | 75.0 | 112 | 1 | 1 | 1 |
| Avery Deer | 1027 | 68.6 | 118 | 1 | 1 | 1 |
| Avery Deer | 1028 | 65.9 | 126 | 1 | 1 | 1 |
| Avery Deer | 1029 | 59.6 | 120 | 1 | 1 | 1 |
| Avery Deer | 1030 | 47.8 | 263 | 1 | 1 | 1 |
| Avery Deer | 1031 | 52.9 | 245 | 1 | 1 | 1 |
| Avery Deer | 1032 | 60.1 | 271 | 1 | 1 | 1 |
| Avery Deer | 1033 | 53.3 | 100 | 1 | 1 | 1 |
| Avery Deer | 1034 | 52.2 | 105 | 1 | 1 | 1 |
| Avery Deer | 1035 | 55.9 | 292 | 1 | 1 | 1 |
| Avery Deer | 1036 | 58.8 | 280 | 1 | 1 | 1 |
| Avery Deer | 1037 | 68.0 | 305 | 1 | 1 | 1 |
| Avery Deer | 1038 | 58.9 | 154 | 1 | 1 | 1 |
| Avery Deer | 1039 | 54.6 | 157 | 1 | 1 | 1 |
| PC | 1040 | 72.2 | 60 | 1 | 1 | 1 |
| PC | 1041 | 67.9 | 52 | 1 | 1 | 1 |
| Hill Marsh | 1042 | 53.2 | 65 | 1 | 1 | 1 |
| Hill Marsh | 1043 | 43.1 | 70 | 1 | 1 | 1 |
| Hill Marsh | 1044 | 57.9 | 95 | 1 | 1 | 1 |
| Hill Marsh | 1045 | 51.9 | 100 | 1 | 1 | 1 |
| Hill Marsh | 1046 | 51.9 | 100 | 1 | 1 | 1 |
| Brushy Creek | 1047 | 42.7 | 62 | 1 | 1 | 1 |
| Brushy Creek | 1048 | 73.9 | 68 | 1 | 1 | 1 |
| Brushy Creek | 1049 | 68.5 | 63 | 1 | 1 | 1 |
| Avery Springhouse | 1050 | 34.2 | 120 | 1 | 1 | 1 |
| Avery Springhouse | 1051 | 23.0 | 120 | 1 | 1 | 1 |
| Avery Springhouse | 1052 | 28.3 | 133 | 1 | 1 | 1 |
| Avery Springhouse | 1053 | 45.3 | 150 | 1 | 1 | 1 |
| Avery Springhouse | 1054 | 17.0 | 170 | 1 | 1 | 1 |
| Avery Springhouse | 1056 | 33.8 | 185 | 1 | 1 | 1 |
| Avery Springhouse | 1057 | 34.1 | 165 | 1 | 1 | 1 |
| Avery Springhouse | 1058 | 23.7 | 128 | 1 | 1 | 1 |
| Avery Springhouse | 1059 | 36.3 | 185 | 1 | 1 | 1 |
| Avery Springhouse | 1060 | 39.5 | 140 | 1 | 1 | 1 |
| Avery Springhouse | 1061 | 47.7 | 150 | 1 | 1 | 0 |
| Avery Springhouse | 1062 | 26.8 | 165 | 0 | 0 | 0 |
| Avery Springhouse | 1063 | 46.5 | 171 | 1 | 1 | 1 |
| Avery Deer | 1064 | 44.6 | 120 | 1 | 1 | 1 |
| Avery Deer | 1065 | 69.5 | 140 | 1 | 1 | 1 |
| Avery Deer | 1066 | 49.0 | 140 | 1 | 0 | 0 |

**R Code and Results**

Green = comments

Blue = code

Black = results (output)

##-------------------------------------------------------------------------------------------------##

## Development and validation of an eDNA assay for central Texas Eurycea salamanders ##

## S1 APPENDIX ##

## ASSAY SENSITIVITY – SALAMANDER-POSITIVE CONTROL ##

library(unmarked)

S1data <- read.csv(file.choose())

S1data

# site = collection site

# sample = collection sample identification number

# tl = salamander total length (mm)

# time = time (minutes) the salamander was held in 250 mL of spring water

# qPCR = result of each qPCR replicate: 0 = no amplification, 1 = amplification

# Format data structure

S1data$tl <- as.numeric(S1data$tl)

S1data$time <- as.numeric(S1data$time)

S1data$qPCR1 <- as.integer(S1data$qPCR1)

S1data$qPCR2 <- as.integer(S1data$qPCR2)

S1data$qPCR3 <- as.integer(S1data$qPCR3)

str(S1data)

## Descriptive Statistics of Salamander Total Length and Time in Water Sample ##

min(S1data[,"tl"])

[1] 17

max(S1data[,"tl"])

[1] 75

mean(S1data[,"tl"])

[1] 49.75472

sd(S1data[,"tl"])

[1] 15.20612

min(S1data[,"time"])

[1] 52

max(S1data[,"time"])

[1] 305

mean(S1data[,"time"])

[1] 139.2453

sd(S1data[,"time"])

[1] 58.96737

## Occupancy Model without Covariates ##

# Identify detection histories

y <- as.matrix(S1data[,5:7])

# Create unmarked dataframe

umf <- unmarkedFrameOccu(y=y)

umf

summary(umf)

unmarkedFrame Object

53 sites

Maximum number of observations per site: 3

Mean number of observations per site: 3

Sites with at least one detection: 52

Tabulation of y observations:

0 1

6 153

# Note that in this analysis each of the 53 samples is considered a “site”

# Occupancy model without covariates

fm1 <- occu(~1 ~1, data=umf)

summary(fm1)

Call:

occu(formula = ~1 ~ 1, data = umf)

Occupancy (logit-scale):

Estimate SE z P(>|z|)

3.95 1.01 3.91 9.13e-05

Detection (logit-scale):

Estimate SE z P(>|z|)

3.93 0.583 6.74 1.57e-11

AIC: 43.57025

Number of sites: 53

optim convergence code: 0

optim iterations: 38

Bootstrap iterations: 0

#Back transform to get estimate of psi

backTransform(fm1, 'state')

Backtransformed linear combination(s) of Occupancy estimate(s)

Estimate SE LinComb (Intercept)

0.981 0.0187 3.95 1

Transformation: logistic

#Back transform to get estimate of p

backTransform(fm1, type = 'det')

Backtransformed linear combination(s) of Detection estimate(s)

Estimate SE LinComb (Intercept)

0.981 0.011 3.93 1

Transformation: logistic
