# Supplementary material for: Development and validation of an environmental DNA assay to detect federally threatened groundwater salamanders in central Texas
Source: PLoS One. 2023 Jul 10;18(7):e0288282. doi: 10.1371/journal.pone.0288282 (PMC10332605; doi:10.1371/journal.pone.0288282)
Supplement: S2 Appendix — (DOCX) [file pone.0288282.s002.docx]

**S2 Appendix. A comparison of psi (ψ) and theta (θ) estimates from spring outlet and downstream sampling locations to inform the assay sensitivity, field control analysis.**

Development and validation of an environmental DNA assay to detect federally threatened groundwater salamanders in central Texas

Zachary C. Adcock, Michelle E. Adcock, Michael R.J. Forstner

Table of Contents:

Purpose 2

Methods 2

Results 3

Discussion 3

References 4

Table A 5

Table B 6

Fig A 7

Data 8

R code and Results 10

**Purpose**

We compared the probability of *Septentriomolge* eDNA occurring at a sampling location (ψ) and the conditional probability of collecting eDNA in a water sample (θ) at upstream and downstream sampling locations in nine headwater creeks (springs). We collected water samples at the spring outlet and at the typical downstream extent of the surface salamander population. Salamanders likely occur in the aquifer upstream of the spring outlet at these sites [e.g., Bendik and Gluesenkamp 2013], but this subsurface habitat is not accessible for visual encounter surveys. Detection of salamander eDNA at the spring outlet would confirm that salamanders occur in the aquifer. Therefore, we were interested in comparing estimates of ψ and θ at the spring outlet to a location just downstream of where salamanders are known to occur in the surface habitat of each headwater creek. We used the results of this analysis to determine if sampling location should be included as a covariate in the multiscale occupancy model to evaluate the sensitivity of the *Septentriomolge* eDNA assay.

**Methods**

We sampled nine headwater creeks at the spring outlet and at the typical downstream extent of the surface *Septentriomolge* population (Table A). The distribution of salamanders at each field control site was previously determined by visual encounter surveys [Adcock 2022, Adcock et al. 2022, Pierce et al. 2010, Pierce et al. 2014]. We collected three 1 L water samples at the spring outlet and three additional 1 L water samples at the downstream sampling location.

For the location model, we treated the spring outlet and spring-downstream sampling locations as separate “sites” (*n* = 18). We fit the model ψ(location)θ(location)*p*(.) to compare the probability of eDNA occurrence at a sampling location (ψ) and the conditional probability of eDNA collection in a water sample (θ) at the spring outlet compared to the typical downstream extent of the salamander population.

- Site = each sampling location (spring outlet and spring-downstream) at nine field control sites for 18 total “sites”
  - The nine field control sites are known to be occupied by one of the three *Septentriomolge* species (i.e., *Eurycea chisholmensis*, *E. naufragia*, and *E. tonkawae*).
    - *E. chisholmensis* sites = Cobbs Spring, Cowan Spring, Twin Springs
    - *E. naufragia* sites = Swinbank Spring
    - *E. tonkawae* sites = Avery Deer Spring, Avery Springhouse Spring, Brushy Creek Spring, Hill Marsh Spring, and PC Spring
- Sample = 1 liter of water
  - We collected three 1 L water samples from each sampling location.
- Replicate = qPCR result
  - We conducted three qPCR replicates per water sample. Therefore, each water sample had three qPCR results to form its detection history.
- Covariates
  - Site = variables that may affect the occurrence of salamander eDNA at a site
    - Sampling location (spring outlet vs spring-downstream)
      - Salamander eDNA is expected at the spring-downstream location because it occurs downstream of a known population of salamanders. It is unknown if salamanders occur upstream of the spring outlet in the aquifer.
  - Sample = variables that may affect the collection of salamander eDNA in a sample
    - Sampling location (spring outlet vs. spring-downstream)
  - Replicate (qPCR) = variables that may affect the detection of eDNA in a qPCR replicate (i.e., may inhibit PCR)
    - none
- Model ψ(location)θ(location)*p*(.) in ‘ednaoccupancy’ notation

| Scale | occModel Component | Covariate(s) |
| --- | --- | --- |
| Site | formulaSite | ~ location |
| Sample | formulaSiteandSample | ~ location |
| qPCR | formulaReplicate | ~ 1 |

**Results**

We detected *Septentriomolge* eDNA at 14 of 18 sites, in 30 of 54 water samples, and in 79 of 162 qPCRs (Table A). We failed to detect eDNA at two spring outlets and two downstream sampling locations. The occurrence probability of *Septentriomolge* eDNA (ψ) and the collection probability of eDNA in a water sample (θ) were not different at spring outlet sampling locations compared to the spring-downstream sampling locations. The 95% CRIs of the slope estimates included zero (Table B), and the 95% CRIs of estimated parameters demonstrated considerable overlap (Fig A). The estimated posterior median probability of occurrence of eDNA (ψ) at the spring outlet was 0.784 (95% CRI: 0.495–0.965) as compared to 0.827 (95% CRI: 0.514–0.993) at the downstream extent of the surface population (Fig A).

**Discussion**

We detected *Septentriomolge* eDNA in at least one sample collected from the spring outlet at seven of nine sites (Table A). There was no difference in the probability of salamander eDNA occurring at the spring outlet compared to the downstream extent of the surface population. There was also no difference in the probability of collecting salamander eDNA in a water sample from the two sampling locations (Table B, Fig A). Therefore, we did not include sampling location as a covariate in the multiscale occupancy model to evaluate the sensitivity of the *Septentriomolge* eDNA assay.

Additionally, these results verify that most sites had subterranean salamanders that would otherwise not be detected by visual encounter surveys. We note that all samples were collected in the winter when some of the salamander population may be subsurface for reproductive activities [Adcock 2022, Bendik 2017, Pierce et al. 2014], and we encourage sampling in other seasons to further understand subterranean occurrence patterns.

**References**

Adcock ZC. The natural history, ecology, and molecular detection of Jollyville Plateau Salamanders (*Eurycea tonkawae*). Ph.D. Dissertation, Texas State University. 2022.

Adcock ZC, MacLaren AR, Jones RM, Villamizar-Gomez A, Wall AE, White IV K, et al. Predicting surface abundance of federally threatened Jollyville Plateau Salamanders (*Eurycea tonkawae*) to inform management activities at a highly modified urban spring. PeerJ 2022;10:e13359.

Bendik NF. Demographics, reproduction, growth, and abundance of Jollyville Plateau salamanders (*Eurycea tonkawae*). Ecol Evol 2017;7:5002–5015.

Bendik NF, Gluesenkamp AG. Body length shrinkage in an endangered amphibian is associated with drought. J Zool 2013;290:35–41.

Pierce BA, Christiansen JL, Ritzer AL, Jones TA. Ecology of Georgetown Salamanders (*Eurycea naufragia*) within the flow of a spring. Southwest Nat 2010;55:291–297.

Pierce BA, McEntire KD, Wall AE. Population size, movement, and reproduction of the Georgetown Salamander, *Eurycea naufragia*. Herpetol Conserv Biol 2014;9:137–145.

Table A. eDNA survey results from field control sites in Williamson County, Texas, USA. The sampling location value corresponds to the distance downstream of the nearest spring outlet.

| Site | *Eurycea* Taxon | Sampling Location | eDNA Surveys | |
| --- | --- | --- | --- | --- |
|  |  |  | Positive Samples / Total Samples | Positive qPCRs / Total qPCRs |
| Avery Deer Spring | *E. tonkawae* | 0 m | 2/3 | 6/9 |
|  |  | 10 m | 2/3 | 5/9 |
| Avery Springhouse Spring | *E. tonkawae* | 0 m | 1/3 | 3/9 |
|  |  | 65 m | 1/3 | 3/9 |
| Brushy Creek Spring | *E. tonkawae* | 0 m | 2/3 | 6/9 |
|  |  | 25 m | 0/3 | 0/9 |
| Cobbs Spring | *E. chisholmensis* | 0 m | 3/3 | 9/9 |
|  |  | 24 m | 3/3 | 8/9 |
| Cowan Spring | *E. chisholmensis* | 0 m | 0/3 | 0/9 |
|  |  | 10 m | 2/3 | 2/9 |
| Hill Marsh Spring | *E. tonkawae* | 0 m | 0/3 | 0/9 |
|  |  | 43 m | 2/3 | 6/9 |
| PC Spring | *E. tonkawae* | 0 m | 3/3 | 7/9 |
|  |  | 3 m | 2/3 | 4/9 |
| Swinbank Spring | *E. naufragia* | 0 m | 2/3 | 5/9 |
|  |  | 24 m | 3/3 | 9/9 |
| Twin Springs | *E. chisholmensis* | 0 m | 2/3 | 6/9 |
|  |  | 7 m | 0/3 | 0/9 |

Table B. Summary of the Bayesian estimates (i.e., posterior mean, median, and 95% credible intervals) of a model comparing parameter estimates from samples at the spring outlet to samples immediately downstream of the surface salamander population.

| Parameter | Mean | Median | 2.5% | 97.5% |
| --- | --- | --- | --- | --- |
| Bayesian estimates of model parameters |  |  |  |  |
| β_ψ_ (intercept) | 0.815 | 0.786 | -0.011 | 1.809 |
| β_ψ_ (spring outlet) | 0.201 | 0.184 | -1.033 | 1.565 |
| α_θ_ (intercept) | 0.463 | 0.463 | -0.094 | 1.035 |
| α_θ_ (spring outlet) | 0.009 | 0.008 | -0.778 | 0.794 |
| δ*_p_* (intercept) | 1.128 | 1.126 | 0.799 | 1.469 |
| Monte Carlo SE of Bayesian estimates |  |  |  |  |
| β_ψ_ (intercept) | 0.0068 | 0.0049 | 0.0062 | 0.0293 |
| β_ψ_ (spring outlet) | 0.0092 | 0.0066 | 0.0187 | 0.0319 |
| α_θ_ (intercept) | 0.0021 | 0.0023 | 0.0039 | 0.0039 |
| α_θ_ (spring outlet) | 0.0029 | 0.0032 | 0.0056 | 0.0054 |
| δ*_p_* (intercept) | 0.0008 | 0.0010 | 0.0020 | 0.0022 |


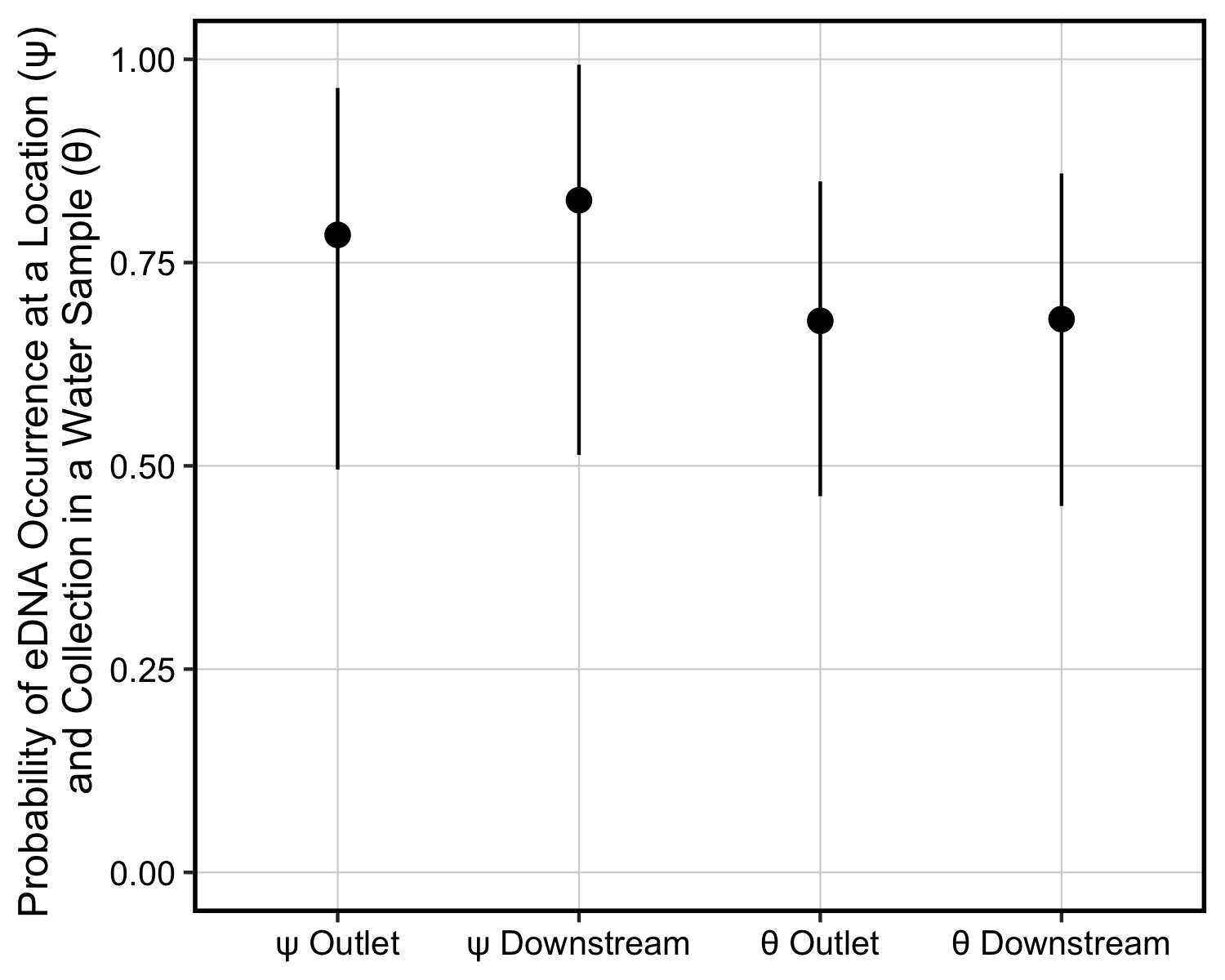


Fig A. Estimated probabilities of *Septentriomolge* eDNA occurrence at a sampling location (ψ) and collection in a water sample (θ) at the spring outlet (Outlet) compared to the downstream extent of the surface population (Downstream).

Symbols represent posterior medians with 95% credible intervals.

**Data**

Table C. Detection histories for each water sample. (locDetectionData)

| site | sample | qPCR1 | qPCR2 | qPCR3 |
| --- | --- | --- | --- | --- |
| Avery Deer down | 1 | 1 | 1 | 1 |
| Avery Deer down | 2 | 1 | 0 | 1 |
| Avery Deer down | 3 | 0 | 0 | 0 |
| Avery Deer up | 1 | 0 | 0 | 0 |
| Avery Deer up | 2 | 1 | 1 | 1 |
| Avery Deer up | 3 | 1 | 1 | 1 |
| Avery Springhouse down | 1 | 1 | 1 | 1 |
| Avery Springhouse down | 2 | 0 | 0 | 0 |
| Avery Springhouse down | 3 | 0 | 0 | 0 |
| Avery Springhouse up | 1 | 1 | 1 | 1 |
| Avery Springhouse up | 2 | 0 | 0 | 0 |
| Avery Springhouse up | 3 | 0 | 0 | 0 |
| Brushy Creek down | 1 | 0 | 0 | 0 |
| Brushy Creek down | 2 | 0 | 0 | 0 |
| Brushy Creek down | 3 | 0 | 0 | 0 |
| Brushy Creek up | 1 | 1 | 1 | 1 |
| Brushy Creek up | 2 | 0 | 0 | 0 |
| Brushy Creek up | 3 | 1 | 1 | 1 |
| Cobbs down | 1 | 1 | 1 | 1 |
| Cobbs down | 2 | 1 | 1 | 1 |
| Cobbs down | 3 | 1 | 1 | 0 |
| Cobbs up | 1 | 1 | 1 | 1 |
| Cobbs up | 2 | 1 | 1 | 1 |
| Cobbs up | 3 | 1 | 1 | 1 |
| Cowan down | 1 | 0 | 1 | 0 |
| Cowan down | 2 | 0 | 0 | 0 |
| Cowan down | 3 | 1 | 0 | 0 |
| Cowan up | 1 | 0 | 0 | 0 |
| Cowan up | 2 | 0 | 0 | 0 |
| Cowan up | 3 | 0 | 0 | 0 |
| Hill Marsh down | 1 | 1 | 1 | 1 |
| Hill Marsh down | 2 | 0 | 0 | 0 |
| Hill Marsh down | 3 | 1 | 1 | 1 |
| Hill Marsh up | 1 | 0 | 0 | 0 |
| Hill Marsh up | 2 | 0 | 0 | 0 |
| Hill Marsh up | 3 | 0 | 0 | 0 |
| PC down | 1 | 1 | 1 | 1 |
| PC down | 2 | 0 | 0 | 1 |
| PC down | 3 | 0 | 0 | 0 |
| PC up | 1 | 1 | 1 | 1 |
| PC up | 2 | 1 | 1 | 1 |
| PC up | 3 | 1 | 0 | 0 |
| Swinbank down | 1 | 1 | 1 | 1 |
| Swinbank down | 2 | 1 | 1 | 1 |
| Swinbank down | 3 | 1 | 1 | 1 |
| Swinbank up | 1 | 0 | 0 | 0 |
| Swinbank up | 2 | 1 | 1 | 1 |
| Swinbank up | 3 | 0 | 1 | 1 |
| Twin down | 1 | 0 | 0 | 0 |
| Twin down | 2 | 0 | 0 | 0 |
| Twin down | 3 | 0 | 0 | 0 |
| Twin up | 1 | 1 | 1 | 1 |
| Twin up | 2 | 0 | 0 | 0 |
| Twin up | 3 | 1 | 1 | 1 |

Table D. Location covariate for each site. (locSurveyData)

| site | location |
| --- | --- |
| PC up | up |
| PC down | down |
| Brushy Creek up | up |
| Brushy Creek down | down |
| Cowan up | up |
| Cowan down | down |
| Twin up | up |
| Twin down | down |
| Swinbank up | up |
| Swinbank down | down |
| Cobbs up | up |
| Cobbs down | down |
| Avery Deer up | up |
| Avery Deer down | down |
| Avery Springhouse up | up |
| Avery Springhouse down | down |
| Hill Marsh up | up |
| Hill Marsh down | down |

**R Code and Results**

Green = comments

Blue = code

Black = results (output)

##-------------------------------------------------------------------------------------------------##

## Development and validation of an eDNA assay for central Texas Eurycea salamanders ##

## S2 APPENDIX ##

## COMPARISON OF eDNA RESULTS FROM SPRING OUTLET AND DOWNSTREAM SAMPLING LOCATIONS ##

library(mvtnorm)

library(pROC)

library(mcmcse)

library(eDNAoccupancy)

library(knitr)

# Read in data

locDetectionData <- read.csv(file.choose())

# site = collection site: treating the spring outlet (up) and downstream extent of the salamander surface population (down) as distinct "sites"

# sample = replicate 1 L water sample per site

# qPCR = result of each qPCR replicate: 0 = no amplification, 1 = amplification

locSurveyData <- read.csv(file.choose())

# site = collection site: treating the spring outlet (up) and downstream extent of the salamander surface population (down) as distinct "sites"

# location = sampling location within the spring run: up = spring outlet and down = the downstream extent of the salamander surface population

### Multiscale Occupancy Model with Sampling Location Covariate ###

# Compute occupancy data matrices

locationDetections = occData(locDetectionData, siteColName = 'site',

sampleColName = 'sample')

# Number of detections per sample

locationDetections$y

[,1] [,2] [,3]

Avery Deer down 3 2 0

Avery Deer up 0 3 3

Avery Springhouse down 3 0 0

Avery Springhouse up 3 0 0

Brushy Creek down 0 0 0

Brushy Creek up 3 0 3

Cobbs down 3 3 2

Cobbs up 3 3 3

Cowan down 1 0 1

Cowan up 0 0 0

Hill Marsh down 3 0 3

Hill Marsh up 0 0 0

PC down 3 1 0

PC up 3 3 1

Swinbank down 3 3 3

Swinbank up 0 3 2

Twin down 0 0 0

Twin up 3 0 3

# Number of PCR replicates per sample

locationDetections$K

[,1] [,2] [,3]

Avery Deer down 3 3 3

Avery Deer up 3 3 3

Avery Springhouse down 3 3 3

Avery Springhouse up 3 3 3

Brushy Creek down 3 3 3

Brushy Creek up 3 3 3

Cobbs down 3 3 3

Cobbs up 3 3 3

Cowan down 3 3 3

Cowan up 3 3 3

Hill Marsh down 3 3 3

Hill Marsh up 3 3 3

PC down 3 3 3

PC up 3 3 3

Swinbank down 3 3 3

Swinbank up 3 3 3

Twin down 3 3 3

Twin up 3 3 3

##-------------------------------------------------------------------------------------------------##

# Fit occupancy model

set.seed(0157)

locmod = occModel(formulaSite = ~ location,

formulaSiteAndSample = ~ location,

formulaReplicate = ~ 1,

detectionMats = locationDetections,

siteData = locSurveyData,

niter = 50000,

niterInterval = 5000,

siteColName = 'site'

)

posteriorSummary(locmod, burnin=5000, mcError=TRUE)

Bayesian estimates of model parameters

Mean 50% 2.5% 97.5%

beta.(Intercept) 0.815 0.786 -0.011 1.809

beta.locationup 0.201 0.184 -1.033 1.565

alpha.(Intercept) 0.463 0.463 -0.094 1.035

alpha.locationup 0.009 0.008 -0.778 0.794

delta.(Intercept) 1.128 1.126 0.799 1.469

Monte Carlo SE of Bayesian estimates

Mean 50% 2.5% 97.5%

beta.(Intercept) 0.0068 0.0049 0.0062 0.0293

beta.locationup 0.0092 0.0066 0.0187 0.0319

alpha.(Intercept) 0.0021 0.0023 0.0039 0.0039

alpha.locationup 0.0029 0.0032 0.0056 0.0054

delta.(Intercept) 0.0008 0.0010 0.0020 0.0022

NULL

# Estimate derived parameters

psiloc = posteriorSummaryOfSiteOccupancy(locmod, burnin=5000)

thetaloc = posteriorSummaryOfSampleOccupancy(locmod, burnin=5000)

ploc = posteriorSummaryOfDetection(locmod, burnin=5000)

# Output estimates of psi posterior medians + CIs

cbind(psi.mean=psiloc$mean, psi.median=psiloc$median, psi.lower=psiloc$lower, psi.upper=psiloc$upper)

psi.mean psi.median psi.lower psi.upper

Avery Deer down 0.808580 0.8266943 0.5135004 0.9933621

Avery Deer up 0.769822 0.7840738 0.4954926 0.9647432

Avery Springhouse down 0.808580 0.8266943 0.5135004 0.9933621

Avery Springhouse up 0.769822 0.7840738 0.4954926 0.9647432

Brushy Creek down 0.808580 0.8266943 0.5135004 0.9933621

Brushy Creek up 0.769822 0.7840738 0.4954926 0.9647432

Cobbs down 0.808580 0.8266943 0.5135004 0.9933621

Cobbs up 0.769822 0.7840738 0.4954926 0.9647432

Cowan down 0.808580 0.8266943 0.5135004 0.9933621

Cowan up 0.769822 0.7840738 0.4954926 0.9647432

Hill Marsh down 0.808580 0.8266943 0.5135004 0.9933621

Hill Marsh up 0.769822 0.7840738 0.4954926 0.9647432

PC down 0.808580 0.8266943 0.5135004 0.9933621

PC up 0.769822 0.7840738 0.4954926 0.9647432

Swinbank down 0.808580 0.8266943 0.5135004 0.9933621

Swinbank up 0.769822 0.7840738 0.4954926 0.9647432

Twin down 0.808580 0.8266943 0.5135004 0.9933621

Twin up 0.769822 0.7840738 0.4954926 0.9647432

SPRING OUTLET PSI = 0.7840738 (0.4954926, 0.9647432)

DOWNSTREAM PSI = 0.8266943 (0.5135004, 0.9933621)

#Output estimates of theta posterior median + CIs

cbind(theta.mean=thetaloc$mean[,1], theta.median=thetaloc$median[,1], theta.lower=thetaloc$lower[,1], theta.upper=thetaloc$upper[,1])

theta.mean theta.median theta.lower theta.upper

Avery Deer down 0.6736573 0.6804836 0.4506866 0.8596543

Avery Deer up 0.6716850 0.6783683 0.4627301 0.8497806

Avery Springhouse down 0.6736573 0.6804836 0.4506866 0.8596543

Avery Springhouse up 0.6716850 0.6783683 0.4627301 0.8497806

Brushy Creek down 0.6736573 0.6804836 0.4506866 0.8596543

Brushy Creek up 0.6716850 0.6783683 0.4627301 0.8497806

Cobbs down 0.6736573 0.6804836 0.4506866 0.8596543

Cobbs up 0.6716850 0.6783683 0.4627301 0.8497806

Cowan down 0.6736573 0.6804836 0.4506866 0.8596543

Cowan up 0.6716850 0.6783683 0.4627301 0.8497806

Hill Marsh down 0.6736573 0.6804836 0.4506866 0.8596543

Hill Marsh up 0.6716850 0.6783683 0.4627301 0.8497806

PC down 0.6736573 0.6804836 0.4506866 0.8596543

PC up 0.6716850 0.6783683 0.4627301 0.8497806

Swinbank down 0.6736573 0.6804836 0.4506866 0.8596543

Swinbank up 0.6716850 0.6783683 0.4627301 0.8497806

Twin down 0.6736573 0.6804836 0.4506866 0.8596543

Twin up 0.6716850 0.6783683 0.4627301 0.8497806

SPRING OUTLET THETA = 0.6783683 (0.4627301, 0.8497806)

DOWNSTREAM THETA = 0.6804836 (0.4506866, 0.8596543)

##-------------------------------------------------------------------------------------------------##

# Sampling Location Plot #

library(ggplot2)

library(forcats)

library(grid)

library(gridBase)

library(gtable)

library(gridExtra)

thetamed <- theta$median[,1]

thetaupper <- theta$upper[,1]

thetalower <- theta$lower[,1]

density <- valSurveyData[, 'density']

dfloc <- data.frame(

trt = factor(c('Psi Up', 'Psi Down', 'Theta Up', 'Theta Down')),

resp = c(0.7840738, 0.8266943, 0.6783683, 0.6804836),

group = factor (c('Up', 'Down', 'Up', 'Down')),

upper = c(0.9647432, 0.9933621, 0.8497806, 0.8596543),

lower = c(0.4954926, 0.5135004, 0.4627301, 0.4506866))

plot.loc <- ggplot(dfloc, aes(x=(fct_inorder(trt)), y=resp)) +

geom_errorbar(aes(ymin=lower, ymax=upper), width=0) +

geom_line() +

geom_point(size=3) +

xlab("Parameters") +

ylab("Probability of eDNA Occurrence at a Location (ψ) \n and Collection in a Water Sample (θ)") +

ylim(0.0, 1.0) +

scale_x_discrete(breaks=c('Psi Up', 'Psi Down', 'Theta Up', 'Theta Down'),

labels=c('ψ Outlet', 'ψ Downstream', 'θ Outlet', 'θ Downstream')) +

theme(axis.text.x = element_text(color = "black", size = 10),

axis.text.y = element_text(color = "black", size = 10),

axis.title.x = element_blank(),

axis.title.y = element_text(color = "black", size = 12),

legend.position = c(.765,0.2),

legend.background = element_rect(fill="white"),

legend.key = element_rect(fill="white"),

panel.background = element_blank(),

panel.grid.major = element_line(size = 0.25, linetype = 'solid', colour = "light gray"),

panel.grid.minor = element_blank(),

panel.border = element_rect(color = "black", fill = NA, size = 1.25))

plot.loc

tiff("FigLoc.tiff", width = 5, height = 4, units = "in", res = 300)

plot.loc

dev.off()
