# Supplementary material for: Development and validation of an environmental DNA assay to detect federally threatened groundwater salamanders in central Texas
Source: PLoS One. 2023 Jul 10;18(7):e0288282. doi: 10.1371/journal.pone.0288282 (PMC10332605; doi:10.1371/journal.pone.0288282)
Supplement: S3 Appendix — (DOCX) [file pone.0288282.s003.docx]

**S3 Appendix. Additional notes, data, R code, and results for the field control to evaluate the sensitivity of the *Septentriomolge* eDNA assay.**

Development and validation of an environmental DNA assay to detect federally threatened groundwater salamanders in central Texas

Zachary C. Adcock, Michelle E. Adcock, Michael R.J. Forstner

Table of Contents:

Additional Notes 2

Data 4

R code and Results 6

Compute data matrices 6

Fit occupancy models and compute model selection indices 7

Trace and autocorrelation plots of top model 13

Estimate derived parameters from top model 14

Compute cumulative probability estimates 15

**Additional Notes**

We estimated the probability of eDNA occurring at a site (ψ), the conditional probability of collecting eDNA in a water sample (θ), and the conditional probability of detecting eDNA in a qPCR replicate (*p*) for *Septentriomolge* salamanders at sites where these salamanders are known to occur. This represents the field control to evaluate the sensitivity of the *Septentriomolge* eDNA assay.

- Site = headwater creek (spring) or well
  - We included 10 sites known to be occupied by one of the three *Septentriomolge* species (i.e., *Eurycea chisholmensis*, *E. naufragia*, and *E. tonkawae*).
    - *E. chisholmensis* sites = Cobbs Spring, Cobbs Well, Cowan Spring, Twin Springs
    - *E. naufragia* sites = Swinbank Spring
    - *E. tonkawae* sites = Avery Deer Spring, Avery Springhouse Spring, Brushy Creek Spring, Hill Marsh Spring, and PC Spring
  - We detected eDNA in at least one water sample at each of the 10 sites.
- Sample = 1 liter of water
  - We collected three 1L water samples from each sampling location.
  - Headwater creeks (springs) were sampled at the spring outlet and at the typical downstream extent of the surface population of salamanders.
  - We found no difference in the probability of occurrence of eDNA at the spring outlet compared to downstream sampling locations (S2 Appendix), and therefore, we treated all six samples as representative of the site.
  - Cobbs Well had a single sampling location.
  - We detected eDNA in 33 of 57 water samples.
- Replicate = qPCR result
  - We conducted three qPCR replicates per water sample. Therefore, each water sample had three qPCR results to form its detection history.
  - We detected eDNA in 88 of 171 qPCRs.
- Covariates
  - Site = none (all sites were known to be occupied by salamanders)
  - Sample = variables that may affect the collection of salamander eDNA in a sample
    - Salamander relative density
      - We estimated the relative density of salamanders (density) at each site from visual encounter surveys conducted on the same day as water sample collection.
    - Water conditions
      - We collected water temperature (temp), pH, dissolved oxygen (do), specific conductance (cond), and flow velocity (flow) at each location of eDNA water sample collection.
      - We used the average of the spring outlet and downstream measures for each water condition parameter from headwater creeks (springs).
      - We removed pH from the analysis because it minimally varied among sites.
  - Replicate (qPCR) = variables that may affect the detection of eDNA in a qPCR replicate (i.e., may inhibit PCR)
    - We used specific conductance as an index of salinity, which is known to negatively influence detection due to PCR inhibition.
- Models
  - We compared eight models:
    - Null = psi(.) theta(.) p(.)
    - 1 = psi(.) theta(density) p(.)
    - 2 = psi(.) theta(temp + do + cond + flow) p(.)
    - 3 = psi(.) theta(density + temp + do + cond + flow) p(.)
    - 4 = psi(.) theta(.) p(cond)
    - 5 = psi(.) theta(density) p(cond)
    - 6 = psi(.) theta(temp + do + cond + flow) p(cond)
    - Full = psi(.) theta(density + temp + do + cond + flow) p(cond)
  - Full model in ‘ednaoccupancy’ notation

| Scale | occModel Component | Covariate(s) |
| --- | --- | --- |
| Site | formulaSite | ~ 1 |
| Sample | formulaSiteandSample | ~ dens + temp + do + cond + flow |
| qPCR | formulaReplicate | ~ cond |

**Data**

Table A. Detection histories for each water sample. (valDetectionData)

| site | sample | pcr1 | pcr2 | pcr3 |
| --- | --- | --- | --- | --- |
| Avery Deer | 1 | 1 | 1 | 1 |
| Avery Deer | 2 | 1 | 0 | 1 |
| Avery Deer | 3 | 0 | 0 | 0 |
| Avery Deer | 4 | 0 | 0 | 0 |
| Avery Deer | 5 | 1 | 1 | 1 |
| Avery Deer | 6 | 1 | 1 | 1 |
| Avery Springhouse | 1 | 1 | 1 | 1 |
| Avery Springhouse | 2 | 0 | 0 | 0 |
| Avery Springhouse | 3 | 0 | 0 | 0 |
| Avery Springhouse | 4 | 1 | 1 | 1 |
| Avery Springhouse | 5 | 0 | 0 | 0 |
| Avery Springhouse | 6 | 0 | 0 | 0 |
| Brushy Creek Spring | 1 | 0 | 0 | 0 |
| Brushy Creek Spring | 2 | 0 | 0 | 0 |
| Brushy Creek Spring | 3 | 0 | 0 | 0 |
| Brushy Creek Spring | 4 | 1 | 1 | 1 |
| Brushy Creek Spring | 5 | 0 | 0 | 0 |
| Brushy Creek Spring | 6 | 1 | 1 | 1 |
| Cobbs | 1 | 1 | 1 | 1 |
| Cobbs | 2 | 1 | 1 | 1 |
| Cobbs | 3 | 1 | 1 | 0 |
| Cobbs | 4 | 1 | 1 | 1 |
| Cobbs | 5 | 1 | 1 | 1 |
| Cobbs | 6 | 1 | 1 | 1 |
| Cobbs Well | 1 | 1 | 1 | 1 |
| Cobbs Well | 2 | 1 | 1 | 1 |
| Cobbs Well | 3 | 1 | 1 | 1 |
| Cowan | 1 | 0 | 1 | 0 |
| Cowan | 2 | 0 | 0 | 0 |
| Cowan | 3 | 1 | 0 | 0 |
| Cowan | 4 | 0 | 0 | 0 |
| Cowan | 5 | 0 | 0 | 0 |
| Cowan | 6 | 0 | 0 | 0 |
| Hill Marsh | 1 | 1 | 1 | 1 |
| Hill Marsh | 2 | 0 | 0 | 0 |
| Hill Marsh | 3 | 1 | 1 | 1 |
| Hill Marsh | 4 | 0 | 0 | 0 |
| Hill Marsh | 5 | 0 | 0 | 0 |
| Hill Marsh | 6 | 0 | 0 | 0 |
| PC | 1 | 1 | 1 | 1 |
| PC | 2 | 0 | 0 | 1 |
| PC | 3 | 0 | 0 | 0 |
| PC | 4 | 1 | 1 | 1 |
| PC | 5 | 1 | 1 | 1 |
| PC | 6 | 1 | 0 | 0 |
| Swinbank | 1 | 1 | 1 | 1 |
| Swinbank | 2 | 1 | 1 | 1 |
| Swinbank | 3 | 1 | 1 | 1 |
| Swinbank | 4 | 0 | 0 | 0 |
| Swinbank | 5 | 1 | 1 | 1 |
| Swinbank | 6 | 0 | 1 | 1 |
| Twin Springs | 1 | 0 | 0 | 0 |
| Twin Springs | 2 | 0 | 0 | 0 |
| Twin Springs | 3 | 0 | 0 | 0 |
| Twin Springs | 4 | 1 | 1 | 1 |
| Twin Springs | 5 | 0 | 0 | 0 |
| Twin Springs | 6 | 1 | 1 | 1 |

Table B. Covariates for each site. (valSurveyData)

| site | temp | do | cond | flow | density |
| --- | --- | --- | --- | --- | --- |
| Avery Deer | 15.1 | 5.70 | 626 | 0.073 | 0.53 |
| Avery Springhouse | 14.8 | 7.55 | 896 | 0.082 | 0.49 |
| Brushy Creek Spring | 16.4 | 5.90 | 879 | 0.014 | 0 |
| Cobbs | 13.6 | 5.05 | 613 | 0.100 | 1.03 |
| Cobbs Well | 13.2 | 5.30 | 622 | 0.002 | 4.00 |
| Cowan | 17.1 | 6.75 | 789 | 0.068 | 0.80 |
| Hill Marsh | 12.6 | 7.70 | 737 | 0.126 | 0.21 |
| PC | 18.6 | 4.05 | 835 | 0.134 | 0.33 |
| Swinbank | 16.9 | 6.85 | 733 | 0.180 | 1.44 |
| Twin Springs | 15.6 | 6.35 | 762 | 0.031 | 0.11 |

**R Code and Results**

Green = comments

Blue = code

Black = results (output)

##-------------------------------------------------------------------------------------------------##

## Development and validation of an eDNA assay for central Texas Eurycea salamanders ##

## S3 APPENDIX ##

## ASSAY SENSITIVITY – KNOWN-OCCUPIED (VALIDATION) SITES CONTROL ##

library(mvtnorm)

library(pROC)

library(mcmcse)

library(eDNAoccupancy)

library(knitr)

# Read in data

valDetectionData <- read.csv(file.choose())

valSurveyData <- read.csv(file.choose())

# Compute occupancy data matrices

valDetections = occData(valDetectionData, siteColName = 'site',

sampleColName = 'sample')

# Number of detections per sample

valDetections$y

[,1] [,2] [,3] [,4] [,5] [,6]

Avery Deer 3 2 0 0 3 3

Avery Springhouse 3 0 0 3 0 0

Brushy Creek Spring 0 0 0 3 0 3

Cobbs 3 3 2 3 3 3

Cobbs Well 3 3 3 NA NA NA

Cowan 1 0 1 0 0 0

Hill Marsh 3 0 3 0 0 0

PC 3 1 0 3 3 1

Swinbank 3 3 3 0 3 2

Twin Springs 0 0 0 3 0 3

# Number of PCR replicates per sample

valDetections$K

[,1] [,2] [,3] [,4] [,5] [,6]

Avery Deer 3 3 3 3 3 3

Avery Springhouse 3 3 3 3 3 3

Brushy Creek Spring 3 3 3 3 3 3

Cobbs 3 3 3 3 3 3

Cobbs Well 3 3 3 0 0 0

Cowan 3 3 3 3 3 3

Hill Marsh 3 3 3 3 3 3

PC 3 3 3 3 3 3

Swinbank 3 3 3 3 3 3

Twin Springs 3 3 3 3 3 3

# Center and scale numeric-valued covariate measurements

valSurveyData.sc = scaleData(valSurveyData)

valSurveyData.sc

site temp do cond flow density

1 Avery Deer -0.1519341 -0.3660101 -1.1816837 -0.14226360 -0.30925737

2 Avery Springhouse -0.3091072 1.2461772 1.4080452 0.01778295 -0.34324170

3 Brushy Creek Spring 0.5291497 -0.1917196 1.2449882 -1.19145765 -0.75954970

4 Cobbs -0.9377999 -0.9324543 -1.3063744 0.33787605 0.11554671

5 Cobbs Well -1.1473642 -0.7145911 -1.2200501 -1.40485305 2.63888296

6 Cowan 0.8958871 0.5490151 0.3817452 -0.23117835 -0.07986317

7 Hill Marsh -1.4617105 1.3768951 -0.1170174 0.80023275 -0.58113199

8 PC 1.6817530 -1.8039068 0.8229583 0.94249635 -0.47917901

9 Swinbank 0.7911050 0.6361604 -0.1553837 1.76051205 0.46388606

10 Twin Springs 0.1100212 0.2004341 0.1227723 -0.88914750 -0.66609280

##-------------------------------------------------------------------------------------------------##

# Fit occupancy models and compute model selection indices

set.seed(0157)

mnull = occModel(formulaSite = ~ 1,

formulaSiteAndSample = ~ 1,

formulaReplicate = ~ 1,

detectionMats = valDetections,

siteData = valSurveyData.sc,

niter = 50000,

niterInterval = 5000,

siteColName = 'site'

)

posteriorSummary(mnull, burnin=5000, mcError=TRUE)

Bayesian estimates of model parameters

Mean 50% 2.5% 97.5%

beta.(Intercept) 1.585 1.546 0.558 2.822

alpha.(Intercept) 0.199 0.199 -0.127 0.526

delta.(Intercept) 1.187 1.184 0.866 1.520

Monte Carlo SE of Bayesian estimates

Mean 50% 2.5% 97.5%

beta.(Intercept) 0.0027 0.0034 0.0056 0.0095

alpha.(Intercept) 0.0008 0.0010 0.0022 0.0020

delta.(Intercept) 0.0008 0.0010 0.0020 0.0022

NULL

posteriorPredictiveLoss(mnull, burnin=5000)

$criterion

[1] 26.62013

$lackOfFit

[1] 15.3618

$predVariance

[1] 11.25833

WAIC(mnull, burnin=5000)

$criterion

[1] 30.80158

$lackOfFit

[1] 26.70429

$predVariance

[1] 4.097286

set.seed(0157)

m1 = occModel(formulaSite = ~ 1,

formulaSiteAndSample = ~ density,

formulaReplicate = ~ 1,

detectionMats = valDetections,

siteData = valSurveyData.sc,

niter = 50000,

niterInterval = 5000,

siteColName = 'site'

)

posteriorSummary(m1, burnin=5000, mcError=TRUE)

Bayesian estimates of model parameters

Mean 50% 2.5% 97.5%

beta.(Intercept) 1.583 1.542 0.566 2.829

alpha.(Intercept) 0.458 0.452 0.056 0.894

alpha.density 1.040 1.014 0.306 1.896

delta.(Intercept) 1.188 1.185 0.869 1.520

Monte Carlo SE of Bayesian estimates

Mean 50% 2.5% 97.5%

beta.(Intercept) 0.0027 0.0034 0.0052 0.0099

alpha.(Intercept) 0.0014 0.0018 0.0030 0.0034

alpha.density 0.0028 0.0036 0.0050 0.0062

delta.(Intercept) 0.0008 0.0010 0.0020 0.0022

NULL

posteriorPredictiveLoss(m1, burnin=5000)

$criterion

[1] 26.5602

$lackOfFit

[1] 15.35838

$predVariance

[1] 11.20182

WAIC(m1, burnin=5000)

$criterion

[1] 30.62293

$lackOfFit

[1] 26.69969

$predVariance

[1] 3.923238

set.seed(0157)

m2 = occModel(formulaSite = ~ 1,

formulaSiteAndSample = ~ temp+do+cond+flow,

formulaReplicate = ~ 1,

detectionMats = valDetections,

siteData = valSurveyData.sc,

niter = 50000,

niterInterval = 5000,

siteColName = 'site'

)

posteriorSummary(m2, burnin=5000, mcError=TRUE)

Bayesian estimates of model parameters

Mean 50% 2.5% 97.5%

beta.(Intercept) 1.587 1.547 0.571 2.831

alpha.(Intercept) 0.321 0.315 -0.051 0.730

alpha.temp -0.151 -0.149 -0.712 0.396

alpha.do -0.636 -0.619 -1.285 -0.104

alpha.cond -0.271 -0.277 -0.802 0.281

alpha.flow 0.385 0.379 -0.040 0.843

delta.(Intercept) 1.182 1.180 0.858 1.519

Monte Carlo SE of Bayesian estimates

Mean 50% 2.5% 97.5%

beta.(Intercept) 0.0027 0.0034 0.0056 0.0098

alpha.(Intercept) 0.0012 0.0013 0.0025 0.0041

alpha.temp 0.0016 0.0020 0.0038 0.0041

alpha.do 0.0021 0.0020 0.0102 0.0035

alpha.cond 0.0016 0.0019 0.0038 0.0046

alpha.flow 0.0014 0.0015 0.0031 0.0048

delta.(Intercept) 0.0009 0.0010 0.0022 0.0022

NULL

posteriorPredictiveLoss(m2, burnin=5000)

$criterion

[1] 27.01847

$lackOfFit

[1] 15.36164

$predVariance

[1] 11.65683

WAIC(m2, burnin=5000)

$criterion

[1] 32.49517

$lackOfFit

[1] 26.76401

$predVariance

[1] 5.731165

set.seed(0157)

m3 = occModel(formulaSite = ~ 1,

formulaSiteAndSample = ~ density+temp+do+cond+flow,

formulaReplicate = ~ 1,

detectionMats = valDetections,

siteData = valSurveyData.sc,

niter = 50000,

niterInterval = 5000,

siteColName = 'site'

)

posteriorSummary(m3, burnin=5000, mcError=TRUE)

Bayesian estimates of model parameters

Mean 50% 2.5% 97.5%

beta.(Intercept) 1.585 1.544 0.553 2.828

alpha.(Intercept) 0.566 0.552 0.069 1.163

alpha.density 0.927 0.878 0.065 2.075

alpha.temp -0.302 -0.300 -0.956 0.297

alpha.do -0.714 -0.699 -1.354 -0.155

alpha.cond -0.023 -0.025 -0.599 0.549

alpha.flow 0.282 0.282 -0.192 0.780

delta.(Intercept) 1.186 1.184 0.866 1.519

Monte Carlo SE of Bayesian estimates

Mean 50% 2.5% 97.5%

beta.(Intercept) 0.0027 0.0033 0.0058 0.0091

alpha.(Intercept) 0.0031 0.0041 0.0070 0.0091

alpha.density 0.0059 0.0084 0.0096 0.0151

alpha.temp 0.0039 0.0048 0.0079 0.0078

alpha.do 0.0040 0.0048 0.0124 0.0080

alpha.cond 0.0038 0.0047 0.0100 0.0068

alpha.flow 0.0029 0.0034 0.0060 0.0090

delta.(Intercept) 0.0009 0.0010 0.0020 0.0022

NULL

posteriorPredictiveLoss(m3, burnin=5000)

$criterion

[1] 26.72676

$lackOfFit

[1] 15.33802

$predVariance

[1] 11.38874

WAIC(m3, burnin=5000)

$criterion

[1] 31.6137

$lackOfFit

[1] 26.72842

$predVariance

[1] 4.885278

set.seed(0157)

m4 = occModel(formulaSite = ~ 1,

formulaSiteAndSample = ~ 1,

formulaReplicate = ~ cond,

detectionMats = valDetections,

siteData = valSurveyData.sc,

niter = 50000,

niterInterval = 5000,

siteColName = 'site'

)

posteriorSummary(m4, burnin=5000, mcError=TRUE)

Bayesian estimates of model parameters

Mean 50% 2.5% 97.5%

beta.(Intercept) 1.585 1.545 0.557 2.833

alpha.(Intercept) 0.208 0.206 -0.117 0.536

delta.(Intercept) 1.168 1.165 0.835 1.517

delta.cond -0.306 -0.302 -0.693 0.062

Monte Carlo SE of Bayesian estimates

Mean 50% 2.5% 97.5%

beta.(Intercept) 0.0027 0.0033 0.0056 0.0090

alpha.(Intercept) 0.0008 0.0010 0.0021 0.0022

delta.(Intercept) 0.0010 0.0012 0.0023 0.0025

delta.cond 0.0011 0.0013 0.0032 0.0027

NULL

posteriorPredictiveLoss(m4, burnin=5000)

$criterion

[1] 27.53027

$lackOfFit

[1] 15.06209

$predVariance

[1] 12.46818

WAIC(m4, burnin=5000)

$criterion

[1] 33.23895

$lackOfFit

[1] 25.70444

$predVariance

[1] 7.53451

set.seed(0157)

m5 = occModel(formulaSite = ~ 1,

formulaSiteAndSample = ~ density,

formulaReplicate = ~ cond,

detectionMats = valDetections,

siteData = valSurveyData.sc,

niter = 50000,

niterInterval = 5000,

siteColName = 'site'

)

posteriorSummary(m5, burnin=5000, mcError=TRUE)

Bayesian estimates of model parameters

Mean 50% 2.5% 97.5%

beta.(Intercept) 1.584 1.546 0.552 2.830

alpha.(Intercept) 0.463 0.457 0.051 0.903

alpha.density 1.034 1.017 0.298 1.895

delta.(Intercept) 1.173 1.170 0.847 1.517

delta.cond -0.295 -0.291 -0.672 0.065

Monte Carlo SE of Bayesian estimates

Mean 50% 2.5% 97.5%

beta.(Intercept) 0.0027 0.0034 0.0058 0.0091

alpha.(Intercept) 0.0014 0.0017 0.0034 0.0034

alpha.density 0.0028 0.0036 0.0049 0.0069

delta.(Intercept) 0.0009 0.0011 0.0023 0.0023

delta.cond 0.0011 0.0013 0.0030 0.0024

NULL

posteriorPredictiveLoss(m5, burnin=5000)

$criterion

[1] 27.03

$lackOfFit

[1] 15.03115

$predVariance

[1] 11.99885

WAIC(m5, burnin=5000)

$criterion

[1] 32.27049

$lackOfFit

[1] 25.63884

$predVariance

[1] 6.631642

set.seed(0157)

m6 = occModel(formulaSite = ~ 1,

formulaSiteAndSample = ~ temp+do+cond+flow,

formulaReplicate = ~ cond,

detectionMats = valDetections,

siteData = valSurveyData.sc,

niter = 50000,

niterInterval = 5000,

siteColName = 'site'

)

posteriorSummary(m6, burnin=5000, mcError=TRUE)

Bayesian estimates of model parameters

Mean 50% 2.5% 97.5%

beta.(Intercept) 1.581 1.539 0.561 2.849

alpha.(Intercept) 0.349 0.341 -0.047 0.797

alpha.temp -0.163 -0.163 -0.726 0.387

alpha.do -0.698 -0.663 -1.514 -0.121

alpha.cond -0.227 -0.236 -0.781 0.380

alpha.flow 0.419 0.405 -0.030 0.955

delta.(Intercept) 1.160 1.156 0.826 1.510

delta.cond -0.305 -0.303 -0.685 0.056

Monte Carlo SE of Bayesian estimates

Mean 50% 2.5% 97.5%

beta.(Intercept) 0.0027 0.0034 0.0055 0.0099

alpha.(Intercept) 0.0017 0.0017 0.0027 0.0051

alpha.temp 0.0017 0.0020 0.0044 0.0041

alpha.do 0.0030 0.0026 0.0114 0.0034

alpha.cond 0.0022 0.0023 0.0036 0.0061

alpha.flow 0.0020 0.0020 0.0031 0.0061

delta.(Intercept) 0.0011 0.0013 0.0023 0.0026

delta.cond 0.0012 0.0013 0.0032 0.0025

NULL

posteriorPredictiveLoss(m6, burnin=5000)

$criterion

[1] 28.04996

$lackOfFit

[1] 15.26747

$predVariance

[1] 12.78248

WAIC(m6, burnin=5000)

$criterion

[1] 34.58062

$lackOfFit

[1] 25.79724

$predVariance

[1] 8.783376

set.seed(0157)

mfull = occModel(formulaSite = ~ 1,

formulaSiteAndSample = ~ density+temp+do+cond+flow,

formulaReplicate = ~ cond,

detectionMats = valDetections,

siteData = valSurveyData.sc,

niter = 50000,

niterInterval = 5000,

siteColName = 'site'

)

posteriorSummary(mfull, burnin=5000, mcError=TRUE)

Bayesian estimates of model parameters

Mean 50% 2.5% 97.5%

beta.(Intercept) 1.589 1.549 0.574 2.834

alpha.(Intercept) 0.582 0.563 0.085 1.162

alpha.density 0.914 0.862 0.047 2.053

alpha.temp -0.303 -0.296 -0.958 0.293

alpha.do -0.749 -0.731 -1.447 -0.157

alpha.cond -0.003 -0.007 -0.589 0.598

alpha.flow 0.300 0.295 -0.187 0.838

delta.(Intercept) 1.167 1.165 0.836 1.515

delta.cond -0.299 -0.297 -0.674 0.061

Monte Carlo SE of Bayesian estimates

Mean 50% 2.5% 97.5%

beta.(Intercept) 0.0027 0.0033 0.0054 0.0094

alpha.(Intercept) 0.0034 0.0041 0.0073 0.0074

alpha.density 0.0062 0.0080 0.0095 0.0137

alpha.temp 0.0040 0.0050 0.0084 0.0081

alpha.do 0.0044 0.0050 0.0125 0.0087

alpha.cond 0.0041 0.0050 0.0116 0.0083

alpha.flow 0.0034 0.0038 0.0059 0.0090

delta.(Intercept) 0.0011 0.0012 0.0023 0.0025

delta.cond 0.0011 0.0012 0.0030 0.0025

NULL

posteriorPredictiveLoss(mfull, burnin=5000)

$criterion

[1] 27.45138

$lackOfFit

[1] 15.11961

$predVariance

[1] 12.33177

WAIC(mfull, burnin=5000)

$criterion

[1] 33.27171

$lackOfFit

[1] 25.6974

$predVariance

[1] 7.574317

| Model Name | Model | Posterior Predictive Loss: Criterion | Posterior Predictive Loss: Lack of Fit | Posterior Predictive Loss: Predictive Variance | WAIC: Criterion | WAIC: Lack of Fit | WAIC: Predictive Variance |
| --- | --- | --- | --- | --- | --- | --- | --- |
| mnull | psi(.) theta(.) p(.) | 26.62 | 15.36 | 11.26 | 30.80 | 26.70 | 4.10 |
| **m1** | **psi(.) theta(dens) p(.)** | **26.56** | **15.36** | **11.20** | **30.62** | **26.70** | **3.93** |
| m2 | psi(.) theta(temp + do + cond + flow) p(.) | 27.02 | 15.36 | 11.66 | 32.50 | 26.76 | 5.73 |
| m3 | psi(.) theta(dens + temp + do + cond + flow) p(.) | 26.73 | 15.34 | 11.39 | 31.61 | 26.73 | 4.89 |
| m4 | psi(.) theta(.) p(cond) | 27.53 | 15.06 | 12.47 | 33.24 | 25.70 | 7.53 |
| m5 | psi(.) theta(dens) p(cond) | 27.03 | 15.03 | 12.00 | 32.27 | 25.64 | 6.63 |
| m6 | psi(.) theta(temp + do + cond + flow) p(cond) | 28.05 | 15.27 | 12.78 | 34.58 | 25.80 | 8.78 |
| mfull | psi(.) theta(dens + temp + do + cond + flow) p(cond) | 27.45 | 15.12 | 12.33 | 33.27 | 25.70 | 7.57 |

****Salamander density model is the top model as indicated by lowest posterior predictive loss and WAIC values****

##-------------------------------------------------------------------------------------------------##

# Rerun top model to overwrite output files for last model run

set.seed(0157)

m1 = occModel(formulaSite = ~ 1,

formulaSiteAndSample = ~ density,

formulaReplicate = ~ 1,

detectionMats = valDetections,

siteData = valSurveyData.sc,

niter = 50000,

niterInterval = 5000,

siteColName = 'site'

)

# Trace plots for top model

plotTrace(m1, c('beta.(Intercept)', 'alpha.(Intercept)', 'alpha.density', 'delta.(Intercept)'), burnin=0)


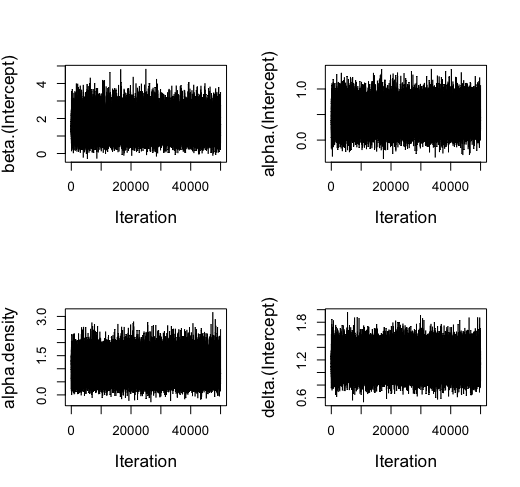


# Autocorrelation plots for top model

plotACF(m1, c('beta.(Intercept)', 'alpha.(Intercept)', 'alpha.density', 'delta.(Intercept)'), burnin=0)


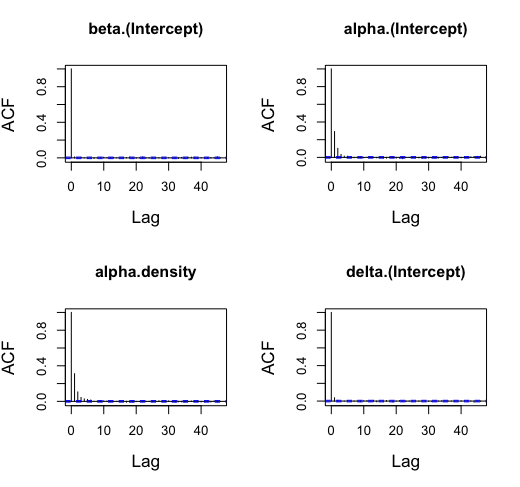


#Estimate derived parameters for top model

psi = posteriorSummaryOfSiteOccupancy(m1, burnin=5000)

theta = posteriorSummaryOfSampleOccupancy(m1, burnin=5000)

p = posteriorSummaryOfDetection(m1, burnin=5000)

#Output estimates of psi posterior median + CIs

cbind(psi.mean=psi$mean, psi.median=psi$median, psi.lower=psi$lower, psi.upper=psi$upper)

psi.mean psi.median psi.lower psi.upper

Avery Deer 0.9161469 0.9384168 0.7143071 0.9976676

Avery Springhouse 0.9161469 0.9384168 0.7143071 0.9976676

Brushy Creek Spring 0.9161469 0.9384168 0.7143071 0.9976676

Cobbs 0.9161469 0.9384168 0.7143071 0.9976676

Cobbs Well 0.9161469 0.9384168 0.7143071 0.9976676

Cowan 0.9161469 0.9384168 0.7143071 0.9976676

Hill Marsh 0.9161469 0.9384168 0.7143071 0.9976676

PC 0.9161469 0.9384168 0.7143071 0.9976676

Swinbank 0.9161469 0.9384168 0.7143071 0.9976676

Twin Springs 0.9161469 0.9384168 0.7143071 0.9976676

#Output estimates of theta posterior median + CIs

cbind(theta.mean=theta$mean[,1], theta.median=theta$median[,1], theta.lower=theta$lower[,1], theta.upper=theta$upper[,1])

theta.mean theta.median theta.lower theta.upper

Avery Deer 0.5534632 0.5541765 0.4186308 0.6828127

Avery Springhouse 0.5396648 0.5406443 0.4036586 0.6708622

Brushy Creek Spring 0.3739050 0.3713584 0.2005496 0.5607180

Cobbs 0.7127109 0.7153534 0.5485603 0.8608514

Cobbs Well 0.9835641 0.9990999 0.8500156 1.0000000

Cowan 0.6434954 0.6447268 0.4994960 0.7803961

Hill Marsh 0.4430484 0.4422568 0.2899156 0.6012994

PC 0.4842153 0.4842635 0.3412082 0.6274475

Swinbank 0.8118607 0.8219029 0.6138666 0.9551473

Twin Springs 0.4095307 0.4079618 0.2466638 0.5810114

#Output estimates of p posterior median + CIs

cbind(p.mean=p$mean[,1], p.median=p$median[,1], p.lower=p$lower[,1], p.upper=p$upper[,1])

p.mean p.median p.lower p.upper

Avery Deer 0.8793425 0.8820699 0.8074632 0.9357733

Avery Springhouse 0.8793425 0.8820699 0.8074632 0.9357733

Brushy Creek Spring 0.8793425 0.8820699 0.8074632 0.9357733

Cobbs 0.8793425 0.8820699 0.8074632 0.9357733

Cobbs Well 0.8793425 0.8820699 0.8074632 0.9357733

Cowan 0.8793425 0.8820699 0.8074632 0.9357733

Hill Marsh 0.8793425 0.8820699 0.8074632 0.9357733

PC 0.8793425 0.8820699 0.8074632 0.9357733

Swinbank 0.8793425 0.8820699 0.8074632 0.9357733

Twin Springs 0.8793425 0.8820699 0.8074632 0.9357733

##-------------------------------------------------------------------------------------------------##

# Plot theta estimates from top model #

library(ggplot2)

library(forcats)

library(grid)

library(gridBase)

library(gtable)

library(gridExtra)

thetamed <- theta$median[,1]

thetaupper <- theta$upper[,1]

thetalower <- theta$lower[,1]

density <- valSurveyData[, 'density']

df <- data.frame(density, thetamed, thetaupper, thetalower)

plot.theta.dens <- ggplot(df, aes(x=density, y=thetamed)) +

geom_errorbar(aes(ymin=thetalower, ymax=thetaupper), width=0) +

geom_point(size=3) +

xlab("Salamander Relative Density (detections/m^2)") +

ylab("Probability of Collecting eDNA in a Sample") +

ylim(0.0, 1.0) +

theme(axis.text.x = element_text(color = "black", size = 10),

axis.text.y = element_text(color = "black", size = 10),

axis.title.x = element_text(color = "black", size = 12),

axis.title.y = element_text(color = "black", size = 12),

panel.background = element_blank(),

panel.grid.major = element_line(size = 0.25, linetype = 'solid', colour = "light gray"),

panel.grid.minor = element_line(size = 0.25, linetype = 'solid', colour = "light gray"),

panel.border = element_rect(color = "black", fill = NA, size = 1.25))

plot.theta.dens

tiff("sal_theta_plot.tiff", width = 5, height = 4, units = "in", res = 300)

plot.theta.dens

dev.off()

##-------------------------------------------------------------------------------------------------##

##-------------------------------------------------------------------------------------------------##

## Cumulative Probability Estimates ##

#Cumulative Theta Probability (theta*)

# Extract data from posterior estimates of theta + CIs to serve as examples of variable salamander densities

theta.prob <- subset(df, select = c(2, 4, 3))

theta.prob <- theta.prob[c(3, 2, 4, 5), ]

theta.prob

thetamed thetalower thetaupper

Brushy Creek Spring 0.3713584 0.2005496 0.5607180

Avery Springhouse 0.5406443 0.4036586 0.6708622

Cobbs 0.7153534 0.5485603 0.8608514

Cobbs Well 0.9990999 0.8500156 1.0000000

# theta* = 1 - (1 - theta)^j (j = number of water samples)

# Brushy Creek Spring (BCS)

BCSmed1 <- 1 - (1 - (theta.prob[1,1]))^1

BCSmed2 <- 1 - (1 - (theta.prob[1,1]))^2

BCSmed3 <- 1 - (1 - (theta.prob[1,1]))^3

BCSmed4 <- 1 - (1 - (theta.prob[1,1]))^4

BCSmed5 <- 1 - (1 - (theta.prob[1,1]))^5

BCSmed6 <- 1 - (1 - (theta.prob[1,1]))^6

BCSmed7 <- 1 - (1 - (theta.prob[1,1]))^7

BCSmed8 <- 1 - (1 - (theta.prob[1,1]))^8

BCSmed9 <- 1 - (1 - (theta.prob[1,1]))^9

BCSmed10 <- 1 - (1 - (theta.prob[1,1]))^10

BCSlow1 <- 1 - (1 - (theta.prob[1,2]))^1

BCSlow2 <- 1 - (1 - (theta.prob[1,2]))^2

BCSlow3 <- 1 - (1 - (theta.prob[1,2]))^3

BCSlow4 <- 1 - (1 - (theta.prob[1,2]))^4

BCSlow5 <- 1 - (1 - (theta.prob[1,2]))^5

BCSlow6 <- 1 - (1 - (theta.prob[1,2]))^6

BCSlow7 <- 1 - (1 - (theta.prob[1,2]))^7

BCSlow8 <- 1 - (1 - (theta.prob[1,2]))^8

BCSlow9 <- 1 - (1 - (theta.prob[1,2]))^9

BCSlow10 <- 1 - (1 - (theta.prob[1,2]))^10

BCSup1 <- 1 - (1 - (theta.prob[1,3]))^1

BCSup2 <- 1 - (1 - (theta.prob[1,3]))^2

BCSup3 <- 1 - (1 - (theta.prob[1,3]))^3

BCSup4 <- 1 - (1 - (theta.prob[1,3]))^4

BCSup5 <- 1 - (1 - (theta.prob[1,3]))^5

BCSup6 <- 1 - (1 - (theta.prob[1,3]))^6

BCSup7 <- 1 - (1 - (theta.prob[1,3]))^7

BCSup8 <- 1 - (1 - (theta.prob[1,3]))^8

BCSup9 <- 1 - (1 - (theta.prob[1,3]))^9

BCSup10 <- 1 - (1 - (theta.prob[1,3]))^10

# Avery Springhouse (AS)

ASmed1 <- 1 - (1 - (theta.prob[2,1]))^1

ASmed2 <- 1 - (1 - (theta.prob[2,1]))^2

ASmed3 <- 1 - (1 - (theta.prob[2,1]))^3

ASmed4 <- 1 - (1 - (theta.prob[2,1]))^4

ASmed5 <- 1 - (1 - (theta.prob[2,1]))^5

ASmed6 <- 1 - (1 - (theta.prob[2,1]))^6

ASmed7 <- 1 - (1 - (theta.prob[2,1]))^7

ASmed8 <- 1 - (1 - (theta.prob[2,1]))^8

ASmed9 <- 1 - (1 - (theta.prob[2,1]))^9

ASmed10 <- 1 - (1 - (theta.prob[2,1]))^10

ASlow1 <- 1 - (1 - (theta.prob[2,2]))^1

ASlow2 <- 1 - (1 - (theta.prob[2,2]))^2

ASlow3 <- 1 - (1 - (theta.prob[2,2]))^3

ASlow4 <- 1 - (1 - (theta.prob[2,2]))^4

ASlow5 <- 1 - (1 - (theta.prob[2,2]))^5

ASlow6 <- 1 - (1 - (theta.prob[2,2]))^6

ASlow7 <- 1 - (1 - (theta.prob[2,2]))^7

ASlow8 <- 1 - (1 - (theta.prob[2,2]))^8

ASlow9 <- 1 - (1 - (theta.prob[2,2]))^9

ASlow10 <- 1 - (1 - (theta.prob[2,2]))^10

ASup1 <- 1 - (1 - (theta.prob[2,3]))^1

ASup2 <- 1 - (1 - (theta.prob[2,3]))^2

ASup3 <- 1 - (1 - (theta.prob[2,3]))^3

ASup4 <- 1 - (1 - (theta.prob[2,3]))^4

ASup5 <- 1 - (1 - (theta.prob[2,3]))^5

ASup6 <- 1 - (1 - (theta.prob[2,3]))^6

ASup7 <- 1 - (1 - (theta.prob[2,3]))^7

ASup8 <- 1 - (1 - (theta.prob[2,3]))^8

ASup9 <- 1 - (1 - (theta.prob[2,3]))^9

ASup10 <- 1 - (1 - (theta.prob[2,3]))^10

# Cobbs Spring (CS)

CSmed1 <- 1 - (1 - (theta.prob[3,1]))^1

CSmed2 <- 1 - (1 - (theta.prob[3,1]))^2

CSmed3 <- 1 - (1 - (theta.prob[3,1]))^3

CSmed4 <- 1 - (1 - (theta.prob[3,1]))^4

CSmed5 <- 1 - (1 - (theta.prob[3,1]))^5

CSmed6 <- 1 - (1 - (theta.prob[3,1]))^6

CSmed7 <- 1 - (1 - (theta.prob[3,1]))^7

CSmed8 <- 1 - (1 - (theta.prob[3,1]))^8

CSmed9 <- 1 - (1 - (theta.prob[3,1]))^9

CSmed10 <- 1 - (1 - (theta.prob[3,1]))^10

CSlow1 <- 1 - (1 - (theta.prob[3,2]))^1

CSlow2 <- 1 - (1 - (theta.prob[3,2]))^2

CSlow3 <- 1 - (1 - (theta.prob[3,2]))^3

CSlow4 <- 1 - (1 - (theta.prob[3,2]))^4

CSlow5 <- 1 - (1 - (theta.prob[3,2]))^5

CSlow6 <- 1 - (1 - (theta.prob[3,2]))^6

CSlow7 <- 1 - (1 - (theta.prob[3,2]))^7

CSlow8 <- 1 - (1 - (theta.prob[3,2]))^8

CSlow9 <- 1 - (1 - (theta.prob[3,2]))^9

CSlow10 <- 1 - (1 - (theta.prob[3,2]))^10

CSup1 <- 1 - (1 - (theta.prob[3,3]))^1

CSup2 <- 1 - (1 - (theta.prob[3,3]))^2

CSup3 <- 1 - (1 - (theta.prob[3,3]))^3

CSup4 <- 1 - (1 - (theta.prob[3,3]))^4

CSup5 <- 1 - (1 - (theta.prob[3,3]))^5

CSup6 <- 1 - (1 - (theta.prob[3,3]))^6

CSup7 <- 1 - (1 - (theta.prob[3,3]))^7

CSup8 <- 1 - (1 - (theta.prob[3,3]))^8

CSup9 <- 1 - (1 - (theta.prob[3,3]))^9

CSup10 <- 1 - (1 - (theta.prob[3,3]))^10

# Cobbs Well (CW)

CWmed1 <- 1 - (1 - (theta.prob[4,1]))^1

CWmed2 <- 1 - (1 - (theta.prob[4,1]))^2

CWmed3 <- 1 - (1 - (theta.prob[4,1]))^3

CWmed4 <- 1 - (1 - (theta.prob[4,1]))^4

CWmed5 <- 1 - (1 - (theta.prob[4,1]))^5

CWmed6 <- 1 - (1 - (theta.prob[4,1]))^6

CWmed7 <- 1 - (1 - (theta.prob[4,1]))^7

CWmed8 <- 1 - (1 - (theta.prob[4,1]))^8

CWmed9 <- 1 - (1 - (theta.prob[4,1]))^9

CWmed10 <- 1 - (1 - (theta.prob[4,1]))^10

CWlow1 <- 1 - (1 - (theta.prob[4,2]))^1

CWlow2 <- 1 - (1 - (theta.prob[4,2]))^2

CWlow3 <- 1 - (1 - (theta.prob[4,2]))^3

CWlow4 <- 1 - (1 - (theta.prob[4,2]))^4

CWlow5 <- 1 - (1 - (theta.prob[4,2]))^5

CWlow6 <- 1 - (1 - (theta.prob[4,2]))^6

CWlow7 <- 1 - (1 - (theta.prob[4,2]))^7

CWlow8 <- 1 - (1 - (theta.prob[4,2]))^8

CWlow9 <- 1 - (1 - (theta.prob[4,2]))^9

CWlow10 <- 1 - (1 - (theta.prob[4,2]))^10

CWup1 <- 1 - (1 - (theta.prob[4,3]))^1

CWup2 <- 1 - (1 - (theta.prob[4,3]))^2

CWup3 <- 1 - (1 - (theta.prob[4,3]))^3

CWup4 <- 1 - (1 - (theta.prob[4,3]))^4

CWup5 <- 1 - (1 - (theta.prob[4,3]))^5

CWup6 <- 1 - (1 - (theta.prob[4,3]))^6

CWup7 <- 1 - (1 - (theta.prob[4,3]))^7

CWup8 <- 1 - (1 - (theta.prob[4,3]))^8

CWup9 <- 1 - (1 - (theta.prob[4,3]))^9

CWup10 <- 1 - (1 - (theta.prob[4,3]))^10

sites <- c(rep("Brushy Creek Spring", times = 10), rep("Avery Springhouse Spring", times = 10),

rep("Cobbs Spring", times = 10), rep("Cobbs Well", times = 10))

samples <- c(rep(1:10, times = 4))

med <- c(BCSmed1, BCSmed2, BCSmed3, BCSmed4, BCSmed5, BCSmed6, BCSmed7, BCSmed8, BCSmed9, BCSmed10,

ASmed1, ASmed2, ASmed3, ASmed4, ASmed5, ASmed6, ASmed7, ASmed8, ASmed9, ASmed10,

CSmed1, CSmed2, CSmed3, CSmed4, CSmed5, CSmed6, CSmed7, CSmed8, CSmed9, CSmed10,

CWmed1, CWmed2, CWmed3, CWmed4, CWmed5, CWmed6, CWmed7, CWmed8, CWmed9, CWmed10)

low <- c(BCSlow1, BCSlow2, BCSlow3, BCSlow4, BCSlow5, BCSlow6, BCSlow7, BCSlow8, BCSlow9, BCSlow10,

ASlow1, ASlow2, ASlow3, ASlow4, ASlow5, ASlow6, ASlow7, ASlow8, ASlow9, ASlow10,

CSlow1, CSlow2, CSlow3, CSlow4, CSlow5, CSlow6, CSlow7, CSlow8, CSlow9, CSlow10,

CWlow1, CWlow2, CWlow3, CWlow4, CWlow5, CWlow6, CWlow7, CWlow8, CWlow9, CWlow10)

up <- c(BCSup1, BCSup2, BCSup3, BCSup4, BCSup5, BCSup6, BCSup7, BCSup8, BCSup9, BCSup10,

ASup1, ASup2, ASup3, ASup4, ASup5, ASup6, ASup7, ASup8, ASup9, ASup10,

CSup1, CSup2, CSup3, CSup4, CSup5, CSup6, CSup7, CSup8, CSup9, CSup10,

CWup1, CWup2, CWup3, CWup4, CWup5, CWup6, CWup7, CWup8, CWup9, CWup10)

df.cum.theta <- data.frame(sites, samples, med, low, up)

df.cum.theta

sites samples med low up

1 Brushy Creek Spring 1 0.3713584 0.2005496 0.5607180

2 Brushy Creek Spring 2 0.6048098 0.3608791 0.8070313

3 Brushy Creek Spring 3 0.7515670 0.4890546 0.9152323

4 Brushy Creek Spring 4 0.8438247 0.5915245 0.9627631

5 Brushy Creek Spring 5 0.9018217 0.6734441 0.9836425

6 Brushy Creek Spring 6 0.9382810 0.7389348 0.9928144

7 Brushy Creek Spring 7 0.9612009 0.7912913 0.9968435

8 Brushy Creek Spring 8 0.9756093 0.8331478 0.9986134

9 Brushy Creek Spring 9 0.9846670 0.8666099 0.9993909

10 Brushy Creek Spring 10 0.9903610 0.8933613 0.9997324

11 Avery Springhouse Spring 1 0.5406443 0.4036586 0.6708622

12 Avery Springhouse Spring 2 0.7889923 0.6443769 0.8916683

13 Avery Springhouse Spring 3 0.9030724 0.7879272 0.9643439

14 Avery Springhouse Spring 4 0.9554758 0.8735322 0.9882642

15 Avery Springhouse Spring 5 0.9795475 0.9245820 0.9961373

16 Avery Springhouse Spring 6 0.9906050 0.9550251 0.9987286

17 Avery Springhouse Spring 7 0.9956844 0.9731796 0.9995815

18 Avery Springhouse Spring 8 0.9980176 0.9840059 0.9998623

19 Avery Springhouse Spring 9 0.9990894 0.9904621 0.9999547

20 Avery Springhouse Spring 10 0.9995817 0.9943121 0.9999851

21 Cobbs Spring 1 0.7153534 0.5485603 0.8608514

22 Cobbs Spring 2 0.9189763 0.7962022 0.9806377

23 Cobbs Spring 3 0.9769369 0.9079976 0.9973058

24 Cobbs Spring 4 0.9934352 0.9584665 0.9996251

25 Cobbs Spring 5 0.9981313 0.9812501 0.9999478

26 Cobbs Spring 6 0.9994681 0.9915356 0.9999927

27 Cobbs Spring 7 0.9998486 0.9961788 0.9999990

28 Cobbs Spring 8 0.9999569 0.9982750 0.9999999

29 Cobbs Spring 9 0.9999877 0.9992213 1.0000000

30 Cobbs Spring 10 0.9999965 0.9996484 1.0000000

31 Cobbs Well 1 0.9990999 0.8500156 1.0000000

32 Cobbs Well 2 0.9999992 0.9775047 1.0000000

33 Cobbs Well 3 1.0000000 0.9966261 1.0000000

34 Cobbs Well 4 1.0000000 0.9994940 1.0000000

35 Cobbs Well 5 1.0000000 0.9999241 1.0000000

36 Cobbs Well 6 1.0000000 0.9999886 1.0000000

37 Cobbs Well 7 1.0000000 0.9999983 1.0000000

38 Cobbs Well 8 1.0000000 0.9999997 1.0000000

39 Cobbs Well 9 1.0000000 1.0000000 1.0000000

40 Cobbs Well 10 1.0000000 1.0000000 1.0000000

# Cumulative Theta Plot

install.packages("wesanderson")

library(wesanderson)

plot.cum.theta <- ggplot(df.cum.theta, aes(x=samples, y=med, color=(fct_inorder(sites)))) +

scale_color_manual(values=wes_palette(name="Moonrise2", n=4, type="discrete")) +

geom_point(size=2.5, position = position_dodge(0.5)) +

geom_errorbar(aes(ymin=low, ymax=up), position = position_dodge(0.5), width=0) +

xlab("Number of Water Samples") +

ylab("Cumulative Collection Probability") +

labs(color = "Sites") +

scale_x_continuous(limits = c(.75, 8.25), breaks = (1:8)) +

ylim(0.0, 1.0) +

geom_hline(yintercept=0.95, linetype = "dashed", color = "black") +

theme(axis.text.x = element_text(color = "black", size = 10),

axis.text.y = element_text(color = "black", size = 10),

axis.title.x = element_text(color = "black", size = 12),

axis.title.y = element_text(color = "black", size = 12),

legend.position = c(.765,0.2),

legend.background = element_rect(fill="white"),

legend.key = element_rect(fill="white"),

panel.background = element_blank(),

panel.grid.major = element_line(size = 0.25, linetype = 'solid', colour = "light gray"),

panel.grid.minor = element_blank(),

panel.border = element_rect(color = "black", fill = NA, size = 1.25))

plot.cum.theta

tiff("cum_theta_plot.tiff", width = 5, height = 4, units = "in", res = 300)

plot.cum.theta

dev.off()

##-------------------------------------------------------------------------------------------------##

# Cumulative p Probability

# p* = 1 - (1 - p)^k (k = number of qpcr replicates)

p1med <- 1 - (1 - (p$median[1,1]))^1

p1low <- 1 - (1 - (p$lower[1,1]))^1

p1up <- 1 - (1 - (p$upper[1,1]))^1

p2med <- 1 - (1 - (p$median[1,1]))^2

p2low <- 1 - (1 - (p$lower[1,1]))^2

p2up <- 1 - (1 - (p$upper[1,1]))^2

p3med <- 1 - (1 - (p$median[1,1]))^3

p3low <- 1 - (1 - (p$lower[1,1]))^3

p3up <- 1 - (1 - (p$upper[1,1]))^3

p4med <- 1 - (1 - (p$median[1,1]))^4

p4low <- 1 - (1 - (p$lower[1,1]))^4

p4up <- 1 - (1 - (p$upper[1,1]))^4

p5med <- 1 - (1 - (p$median[1,1]))^5

p5low <- 1 - (1 - (p$lower[1,1]))^5

p5up <- 1 - (1 - (p$upper[1,1]))^5

qpcrs <- c(1, 2, 3, 4, 5)

pmed <- c(p1med, p2med, p3med, p4med, p5med)

plow <- c(p1low, p2low, p3low, p4low, p5low)

pup <- c(p1up, p2up, p3up, p4up, p5up)

df.p <- data.frame(qpcrs, pmed, plow, pup)

df.p

qpcrs pmed plow pup

1 1 0.8820699 0.8074632 0.9357733

2 2 0.9860925 0.9629296 0.9958749

3 3 0.9983599 0.9928626 0.9997351

4 4 0.9998066 0.9986258 0.9999830

5 5 0.9999772 0.9997354 0.9999989

# Cumulative p Plot

plot.cum.p <- ggplot(df.p, aes(x=qpcrs, y=pmed)) +

geom_errorbar(aes(ymin=plow, ymax=pup), width=0) +

geom_point(size=2.5) +

scale_x_continuous(limits = c(1, 4), breaks = (1:4)) +

xlab("Number of qPCR Replicates") +

ylab("Cumulative Detection Probability") +

ylim(0.0, 1.0) +

geom_hline(yintercept=0.95, linetype = "dashed", color = "black") +

theme(axis.text.x = element_text(color = "black", size = 10),

axis.text.y = element_text(color = "black", size = 10),

axis.title.x = element_text(color = "black", size = 12),

axis.title.y = element_text(color = "black", size = 12),

panel.background = element_blank(),

panel.grid.major = element_line(size = 0.25, linetype = 'solid', colour = "light gray"),

panel.grid.minor = element_blank(),

panel.border = element_rect(color = "black", fill = NA, size = 1.25))

plot.cum.p

tiff("cum_p_plot.tiff", width = 5, height = 4, units = "in", res = 300)

plot.cum.p

dev.off()
