# Supplementary material for: Development and validation of an environmental DNA assay to detect federally threatened groundwater salamanders in central Texas
Source: PLoS One. 2023 Jul 10;18(7):e0288282. doi: 10.1371/journal.pone.0288282 (PMC10332605; doi:10.1371/journal.pone.0288282)
Supplement: S4 Appendix — (DOCX) [file pone.0288282.s004.docx]

**S4 Appendix. Additional notes, data, R code, and results for *Septentriomolge* visual encounter surveys to compare to the *Septentriomolge* eDNA assay.**

Development and validation of an environmental DNA assay to detect federally threatened groundwater salamanders in central Texas

Zachary C. Adcock, Michelle E. Adcock, Michael R.J. Forstner

Table of Contents:

Additional Notes 2

Data 3

R code and Results 4

**Additional Notes**

We conducted visual encounter surveys to provide a comparison of site occupancy estimates between standard surveys and eDNA. We estimated the probability of salamanders occurring at a site (ψ) and the probability of detecting salamanders in a visual encounter survey (*p*) at sites where these salamanders are known to occur.

- Site = headwater creek (spring) or well
  - We conducted visual encounter surveys at 10 sites known to be occupied by one of the three *Septentriomolge* species (i.e., *Eurycea chisholmensis*, *E. naufragia*, and *E. tonkawae*).
    - *E. chisholmensis* sites = Cobbs Spring, Cobbs Well, Cowan Spring, Twin Springs
    - *E. naufragia* sites = Swinbank Spring
    - *E. tonkawae* sites = Avery Deer Spring, Avery Springhouse Spring, Brushy Creek Spring, Hill Marsh Spring, and PC Spring
  - Each of these sites were included in the eDNA field control.
  - We detected salamanders in at least one visual encounter survey at nine of the 10 sites.
    - We did not detect salamanders at Brushy Creek Spring.
- Replicate = visual encounter survey
  - All visual encounter surveys followed USFWS survey protocol and consisted of searching in and under potential cover objects for salamanders.
  - We conducted visual encounter surveys at each of these sites every month or every other month prior to eDNA sampling and on the same day as eDNA sampling.
  - We included detection/nondetection data from visual encounter surveys conducted on the day of eDNA sample collection and surveys from the three previous months which corresponds to the early portion of the *Septentriomolge* breeding season.
  - We assumed demographic closure over this timeframe, resulting in 2–4 temporal survey replicates at each site.
  - We detected salamanders in 26 of 30 visual encounter surveys.
    - We detected salamanders during every survey at eight sites.
    - We detected salamanders during two of four surveys at Twin Springs.
    - We did not detect salamanders in the two surveys conducted at Brushy Creek Spring.
- Model
  - Null = psi(.) p(.)

**Data**

Table A. Site and results of visual encounter surveys conducted from October 2019 to January 2020. ‘0’ designates no salamanders detected and ‘1’ designates salamanders detected. (VESdata)

| site | Oct-19 | Nov-19 | Dec-19 | Jan-20 |
| --- | --- | --- | --- | --- |
| Avery Deer |  | 1 |  | 1 |
| Avery Springhouse |  | 1 |  | 1 |
| Hill Marsh |  | 1 |  | 1 |
| PC | 1 |  | 1 |  |
| Brushy Creek | 0 |  | 0 |  |
| Cobbs Spring | 1 | 1 | 1 | 1 |
| Cobbs Well | 1 | 1 | 1 | 1 |
| Cowan | 1 | 1 | 1 | 1 |
| Swinbank | 1 | 1 | 1 | 1 |
| Twin | 0 | 1 | 1 | 0 |

**R Code and Results**

Green = comments

Blue = code

Black = results (output)

##-------------------------------------------------------------------------------------------------##

## Development and validation of an eDNA assay for central Texas Eurycea salamanders ##

## S4 APPENDIX ##

## ASSAY SENSITIVITY - COMPARATIVE VISUAL ENCOUNTER SURVEYS (VES) AT SEPTENTRIOMOLGE-OCCUPIED SITES ##

library(unmarked)

VESdata <- read.csv(file.choose())

VESdata

#site = survey site

#remaining columns = month_year of survey with result: 0 = no salamanders detected, 1 = salamanders detected

# Format data structure

VESdata$oct2019 <- as.integer(VESdata$oct2019)

VESdata$nov2019 <- as.integer(VESdata$nov2019)

VESdata$dec2019 <- as.integer(VESdata$dec2019)

VESdata$jan2020 <- as.integer(VESdata$jan2020)

str(VESdata)

## Occupancy Model without Covariates ##

# Identify detection histories

VESy <- as.matrix(VESdata[,2:5])

# Create unmarked dataframe

VESumf <- unmarkedFrameOccu(y=VESy)

VESumf

summary(VESumf)

unmarkedFrame Object

10 sites

Maximum number of observations per site: 4

Mean number of observations per site: 3

Sites with at least one detection: 9

Tabulation of y observations:

0 1 <NA>

4 26 10

# Occupancy model without covariates

VESm <- occu(~1 ~1, data=VESumf)

summary(VESm)

Call:

occu(formula = ~1 ~ 1, data = VESumf)

Occupancy (logit-scale):

Estimate SE z P(>|z|)

2.25 1.11 2.03 0.0428

Detection (logit-scale):

Estimate SE z P(>|z|)

2.52 0.752 3.35 0.000818

AIC: 24.81517

Number of sites: 10

optim convergence code: 0

optim iterations: 14

Bootstrap iterations: 0

#Back transform to get estimate of psi

backTransform(VESm, 'state')

Backtransformed linear combination(s) of Occupancy estimate(s)

Estimate SE LinComb (Intercept)

0.905 0.0957 2.25 1

Transformation: logistic

#Back transform to get estimate of p

backTransform(VESm, type = 'det')

Backtransformed linear combination(s) of Detection estimate(s)

Estimate SE LinComb (Intercept)

0.925 0.052 2.52 1

Transformation: logistic
