# Supplementary material for: Development and validation of an environmental DNA assay to detect federally threatened groundwater salamanders in central Texas
Source: PLoS One. 2023 Jul 10;18(7):e0288282. doi: 10.1371/journal.pone.0288282 (PMC10332605; doi:10.1371/journal.pone.0288282)
Supplement: S5 Appendix — (DOCX) [file pone.0288282.s005.docx]

**S5 Appendix. Application of the *Septentriomolge* eDNA assay at field sites with rare salamander detections and second order creeks downstream of occupied sites.**

Development and validation of an environmental DNA assay to detect federally threatened groundwater salamanders in central Texas

Zachary C. Adcock, Michelle E. Adcock, Michael R.J. Forstner

Table of Contents:

Purpose 2

Methods 2

Results 2

Discussion 2

References 3

Table A 4

Fig A 5

**Purpose**

We applied the *Septentriomolge* eDNA assay at sites with rare salamander detections and in second order creeks downstream of known-occupied sites (Fig A). Our intention was to test the assay at sites with an unknown occupancy status (i.e., rare detection sites) and to explore potential downstream transport of eDNA (i.e., second order creeks).

**Methods**

We collected water samples from five application sites with historically rare detections of a *Septentriomolge* taxon (Table A, Fig A). If salamanders occupy these sites, then they either have very low detectability in the surveyable portions of the spring, or salamanders are absent from surveyable portions but exist in un-surveyable areas (i.e., subterranean). It is also possible that salamanders are usually absent from these sites and that rare recolonization events occur.

Second order creeks occurred downstream of *Septentriomolge*-occupied springs (Table A, Fig A). We sampled Brushy Creek approximately 4.5 km downstream of at least four occupied springs, Lake Creek approximately 3.4 km downstream of PC Spring, and North Fork San Gabriel River just below its confluence with Swinbank Spring which is approximately 70 m downstream of the Swinbank Spring outlet. There are no records of salamander detections in the second order creeks [Adcock et al. 2022].

We collected three 1 L water samples and one 1 L negative control at each application site. We collected water samples at the spring outlet for rare detection sites and at the thalweg of second order creeks. Laboratory methods followed the same protocols as described for the field control sites in the manuscript.

**Results**

We did not detect *Septentriomolge* eDNA at any of the sites with rare detections (*n* = 5) or second order creeks (*n* = 3). These included a total of 24 water samples and 72 qPCR replicates (Table A). We were unable to fully filter water samples from one of the rare detection sites, Bat Well Cave, and all the second order creeks due to suspended particulates clogging the filters. The average filtered water volume per sample at these sites was: Bat Well Cave = 0.55 L, North Fork San Gabriel River = 0.33 L, Brushy Creek = 0.37 L, and Lake Creek = 0.27 L, as compared to 1 L from all other samples.

**Discussion**

It is not clear if the eight treatment sites were unoccupied or if they were occupied but salamander eDNA was not detected. We had issues with suspended particulates clogging filters and causing incomplete filtering at all second order creek sites and at Bat Well Cave. The amount of water successfully filtered from each second order creek totaled < 1.1 L and < 1.7 L at Bat Well Cave, considerably less than the amount of water that we estimated is necessary to achieve 0.95 cumulative probability of eDNA occurrence at most sites (see Fig 3 in manuscript and S3 Appendix). Future work in similar systems will need to remove suspended particulates before eDNA filtering [Beng and Corlett 2020, Hunter et al. 2019].

We confirmed *Septentriomolge* populations upstream of all second order creek sites, yet we did not detect eDNA in any second order creek samples. It is possible that DNA degradation during downstream transit or eDNA dilution contributed to these negative results. However, the most plausible explanation is that inadequate water sample size, due to incomplete filtering, prevented detection.

**References**

Adcock ZC, MacLaren AR, Jones RM, Villamizar-Gomez A, Wall AE, White IV K, et al. Predicting surface abundance of federally threatened Jollyville Plateau Salamanders (*Eurycea tonkawae*) to inform management activities at a highly modified urban spring. PeerJ 2022;10:e13359.

Beng KC, Corlett RT. Applications of environmental DNA (eDNA) in ecology and conservation: opportunities, challenges, and prospects. Biodivers Conserv 2020;29:2089–2121.

Chippindale PT, Price AH, Wiens JJ, Hillis DM. Phylogenetic relationships and systematic revision of central Texas hemidactyliine plethodontid salamanders. Herpetological Monographs 2000;14:1–80.

Devitt TJ, Wright AM, Canatella DC, Hillis DM. Species delimitation in endangered groundwater salamanders: Implications for aquifer management and biodiversity conservation. Proc Natl Acad Sci U S A 2019;116:2624–2633.

Hunter ME, Ferrante JA, Meigs-Friend G, Ulmer A. Improving eDNA yield and inhibitor reduction through increased water volumes and multi-filter isolation techniques. Sci Rep 2019;9:5259.

Sweet SS. A distributional analysis of epigean populations of *Eurycea* *neotenes* in central Texas, with comments on the origin of troglobitic populations. Herpetologica 1982;38:430–444.

The University of Texas at Austin–Texas Natural History Collections. TNHC Herpetology Collection. 2018 Nov 13 [cited 2021 Jan 23]. Available from: <http://ipt.vertnet.org:8080/ipt/resource.do?r=tnhc_herps>.

Table A. Site information and eDNA survey results for sites with rare detections of a *Septentriomolge* taxon and second order creeks that occur downstream of *Septentriomolge*-occupied springs in Williamson County, Texas, USA. We include the expected taxon based on Devitt et al. (2019) and the total number of documented *Septentriomolge* detections from each spring at the time of eDNA collection. The sampling location value corresponds to the distance downstream of the nearest spring outlet for rare detection sites and the distance downstream of the nearest *Septentriomolge*-occupied spring outlet for second order creeks.

| Application Category | Site | *Eurycea* Taxon | Recorded Detections | Sampling Location | eDNA Surveys | |
| --- | --- | --- | --- | --- | --- | --- |
|  |  |  |  |  | Positive Samples / Total Samples | Positive qPCRs / Total qPCRs |
| Rare detection | Bat Well Cave | *E. chisholmensis* | 1^a^ | -- | 0/3 | 0/9 |
| Rare detection | San Gabriel Springs–east | *E. tonkawae* | 0 | 0 m | 0/3 | 0/9 |
| Rare detection | San Gabriel Springs–middle | *E. tonkawae* | 1^b^ | 0 m | 0/3 | 0/9 |
| Rare detection | San Gabriel Springs–west | *E. tonkawae* | 1^c^ | 0 m | 0/3 | 0/9 |
| Rare detection | San Gabriel Springs–Beaver Spring | *E. tonkawae* | 2^d^ | 0 m | 0/3 | 0/9 |
| Second order creek | Brushy Creek | *E. tonkawae* | 0 | 4.5 km | 0/3 | 0/9 |
| Second order creek | Lake Creek | *E. tonkawae* | 0 | 3.4 km | 0/3 | 0/9 |
| Second order creek | North Fork San Gabriel River | *E. naufragia* | 0 | 70 m | 0/3 | 0/9 |

^a^TNHC 54573 [UT-TNHC 2021]

^b^[Chippindale et al. 2000]

^c^[Sweet 1982]

^d^TNHC 73001–73002 [UT-TNHC 2021]


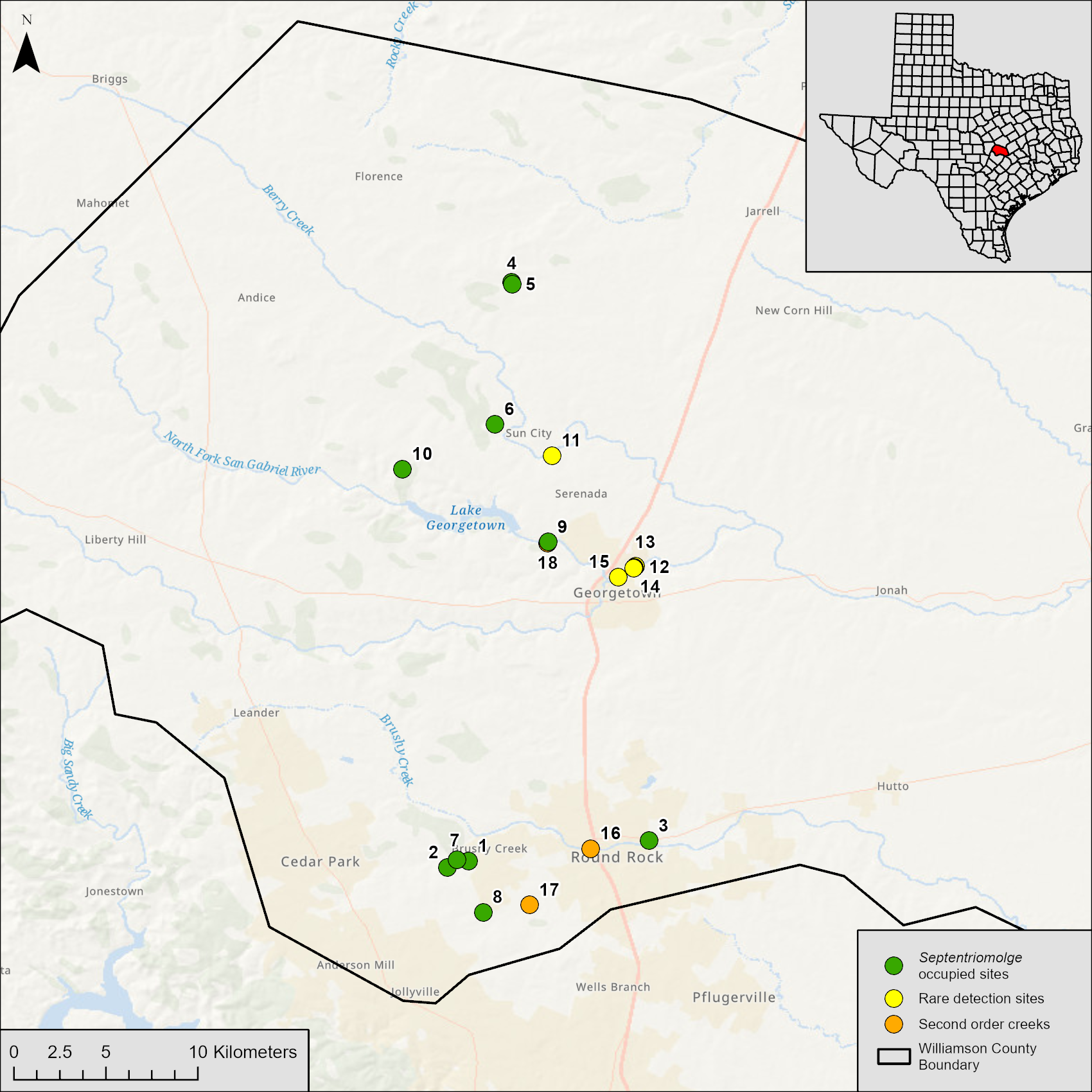


Fig A. eDNA sampling locations in Williamson County, Texas, USA.

Green circles denote field control sites that are known to be occupied by either *Eurycea chisholmensis*, *E. naufragia*, or *E. tonkawae* (*Septentriomolge* clade): 1) Avery Deer Spring, 2) Avery Springhouse Spring, 3) Brushy Creek Spring, 4) Cobbs Spring, 5) Cobbs Well, 6) Cowan Spring, 7) Hill Marsh Spring, 8) PC Spring, 9) Swinbank Spring, 10) Twin Springs. Yellow circles denote field application sites that have rare detections: 11) Bat Well Cave, 12) San Gabriel Springs–east, 13) San Gabriel Springs–middle, 14) San Gabriel Springs–west, 15) San Gabriel Springs–Beaver Spring. Orange circles denote field application sites that are second order creeks downstream of a known *Septentriomolge* population(s): 16) Brushy Creek, 17) Lake Creek, 18) North Fork San Gabriel River. Spatial file sourced from the USGS National Map Viewer.
