# Supplementary material for: Development and validation of an environmental DNA assay to detect federally threatened groundwater salamanders in central Texas
Source: PLoS One. 2023 Jul 10;18(7):e0288282. doi: 10.1371/journal.pone.0288282 (PMC10332605; doi:10.1371/journal.pone.0288282)
Supplement: S1 Table — Non-target amphibians have a distributional overlap with Septentriomolge in Travis, Williamson, or Bell counties, Texas, USA [Dixon 2013]. Ambystoma tigrinum is not currently known to overlap in distribution but has potential for introduction because it is commonly used as fish bait [Dixon 2013]. Eleutherodactylus coqui, Eleutherodactylus planirostris, Pseudacris feriarum, and Scaphiopus hurterii are not known to overlap in distribution, but these sequences were used as surrogates or to supplement data for the overlapping genus. (DOCX) [file pone.0288282.s006.docx]

**S1 Table. Classification and GenBank accession number for target *Septentriomolge* and non-target amphibian mitochondrial DNA *cytochrome b* sequences used to design the *Septentriomolge*-specific qPCR assay.** Non-target amphibians have a distributional overlap with *Septentriomolge* in Travis, Williamson, or Bell counties, Texas, USA [Dixon 2013]. *Ambystoma tigrinum* is not currently known to overlap in distribution but has potential for introduction because it is commonly used as fish bait [Dixon 2013]. *Eleutherodactylus coqui*, *Eleutherodactylus planirostris*, *Pseudacris feriarum*, and *Scaphiopus hurterii* are not known to overlap in distribution, but these sequences were used as surrogates or to supplement data for the overlapping genus.

| Panel Group | Species | GenBank Accession Number |
| --- | --- | --- |
| Target *Eurycea* | *Eurycea chisholmensis* | AY014841 |
| Target *Eurycea* | *Eurycea naufragia* | AY014843, JQ920627 |
| Target *Eurycea* | *Eurycea tonkawae* | AY014842, HQ713591, HQ713592, HQ713593, HQ713594, JQ920626 |
| Overlapping caudate | *Ambystoma texanum* | EF036642, EF036644, EF036648, EF036662, EF036664 |
| Overlapping caudate | *Ambystoma tigrinum* | EF036665, EF036666, EF036667, U36419 |
| Overlapping caudate | *Plethodon albagula* | DQ994905, DQ994906, DQ994907, DQ994908, DQ994909, DQ994910, JF504320 |
| Overlapping anuran | *Acris blanchardi* | EF988098, EF988099, EF988100, EF988101, EF988102 |
| Overlapping anuran | *Anaxyrus* (*Bufo*) *debilis* | AY010165, L10970 |
| Overlapping anuran | *Anaxyrus* (*Bufo*) *punctatus* | DQ85631, DQ85632, DQ85633, DQ85634, EU938445 |
| Overlapping anuran | *Anaxyrus* (*Bufo*) *speciosus* | L10980 |
| Overlapping anuran | *Anaxyrus* (*Bufo*) *woodhousii* | AY288056, AY288057, AY288058, AY288061, AY288064 |
| Overlapping anuran | *Craugastor augusti* | JX564870 |
| Overlapping anuran | *Eleutherodactylus coqui* | EF636951, EF636952, EF636953, EF636954, EF636955 |
| Overlapping anuran | *Eleutherodactylus cystignathoides* | no sequences available |
| Overlapping anuran | *Eleutherodactylus marnockii* | no sequences available |
| Overlapping anuran | *Eleutherodactylus planirostris* | HQ831557, HQ831560, HQ831562, HQ831564, HQ831567 |
| Overlapping anuran | *Gastrophryne carolinensis* | no sequences available |
| Overlapping anuran | *Gastrophryne olivacea* | JX564865 |
| Overlapping anuran | *Hyla chrysoscelis* | AY830968, AY830978, AY830982, AY830984, AY830999 |
| Overlapping anuran | *Hyla cinerea* | AY549380, AY843846, FJ226874, KJ536188, KJ536189 |
| Overlapping anuran | *Hyla versicolor* | AY830973, AY830995, AY830998, AY831010, AY8301014 |
| Overlapping anuran | *Incilius* (*Bufo*) *nebulifer* | AY008174, AY008206, HM563960, HM563961, HQ290525 |
| Overlapping anuran | *Lithobates* (*Rana*) *berlandieri* | KX269301 |
| Overlapping anuran | *Lithobates* (*Rana*) *catesbeiana* | AF205089, KX344485, KX344486, KX344487, KX344488 |
| Overlapping anuran | *Lithobates* (*Rana*) *sphenocephala* | KX269321 |
| Overlapping anuran | *Pseudacris clarkii* | KJ536214, KJ536216 |
| Overlapping anuran | *Pseudacris feriarum* | KJ536218, KJ536219, KJ536220, KJ536221 |
| Overlapping anuran | *Pseudacris fouquettei* | KJ536226, KJ536227 |
| Overlapping anuran | *Pseudacris streckeri* | AY210861, KJ536206, KJ536207 |
| Overlapping anuran | *Scaphiopus couchii* | AY236791, AY236792, JX564894 |
| Overlapping anuran | *Scaphiopus hurterii* | AY236793 |

**References**

Dixon JR. Amphibians & reptiles of Texas: with keys, taxonomic synopses, bibliography, and distribution maps. 3^rd^ ed. College Station: Texas A&M University Press; 2013.
