# Supplementary material for: Development and validation of an environmental DNA assay to detect federally threatened groundwater salamanders in central Texas
Source: PLoS One. 2023 Jul 10;18(7):e0288282. doi: 10.1371/journal.pone.0288282 (PMC10332605; doi:10.1371/journal.pone.0288282)
Supplement: S2 Table — Non-target amphibians have a distributional overlap with Septentriomolge in Travis, Williamson, or Bell counties, Texas, USA [Dixon 2013]. Ambystoma tigrinum is not currently known to overlap in distribution but has potential for introduction because it is commonly used as fish bait [Dixon 2013]. (DOCX) [file pone.0288282.s007.docx]

**S2 Table. Classification, collector number, collection county (Texas, USA), and results for target *Septentriomolge* and non-target amphibian tissue samples used to test the specificity of the *Septentriomolge* qPCR assay.** Non-target amphibians have a distributional overlap with *Septentriomolge* in Travis, Williamson, or Bell counties, Texas, USA [Dixon 2013]. *Ambystoma tigrinum* is not currently known to overlap in distribution but has potential for introduction because it is commonly used as fish bait [Dixon 2013].

| Panel Group | Species | Collector Number | Collection County | Positive qPCRs / Total qPCRs |
| --- | --- | --- | --- | --- |
| Target *Septentriomolge* | *Eurycea chisholmensis* | ZCA1113 | Williamson | 3/3 |
| Target *Septentriomolge* | *Eurycea chisholmensis* | ZCA1118 | Williamson | 3/3 |
| Target *Septentriomolge* | *Eurycea tonkawae* | ZCA0994 | Williamson | 3/3 |
| Target *Septentriomolge* | *Eurycea tonkawae* | MF38033 | Williamson | 3/3 |
| Target *Septentriomolge* | *Eurycea tonkawae* | MF37192 | Travis | 3/3 |
| Target *Septentriomolge* | *Eurycea tonkawae* | MF20933 | Travis | 3/3 |
| Overlapping caudate | *Ambystoma texanum* | MF27865 | Bastrop | 0/3 |
| Overlapping caudate | *Ambystoma texanum* | MF27866 | Bastrop | 0/3 |
| Overlapping caudate | *Ambystoma tigrinum* | MF32549 | Bastrop | 0/3 |
| Overlapping caudate | *Ambystoma tigrinum* | MF32550 | Bastrop | 0/3 |
| Overlapping caudate | *Plethodon albagula* | MF2494 | Bandera | 0/3 |
| Overlapping caudate | *Plethodon albagula* | MF20016 | Edwards | 0/3 |
| Overlapping anuran | *Acris blanchardi* | MF22042 | Travis | 0/3 |
| Overlapping anuran | *Acris blanchardi* | MF22049 | Travis | 0/3 |
| Overlapping anuran | *Anaxyrus* (*Bufo*) *debilis* | MF22959 | Presidio | 0/3 |
| Overlapping anuran | *Anaxyrus* (*Bufo*) *debilis* | MF22960 | Presidio | 0/3 |
| Overlapping anuran | *Anaxyrus* (*Bufo*) *punctatus* | MF38309 | Val Verde | 0/3 |
| Overlapping anuran | *Anaxyrus* (*Bufo*) *punctatus* | MF4208 | Edwards | 0/3 |
| Overlapping anuran | *Anaxyrus* (*Bufo*) *speciosus* | MF38227 | Terrell | 0/3 |
| Overlapping anuran | *Anaxyrus* (*Bufo*) *speciosus* | MF38267 | Terrell | 0/3 |
| Overlapping anuran | *Anaxyrus* (*Bufo*) *woodhousii* | MF26120 | Henderson | 0/3 |
| Overlapping anuran | *Anaxyrus* (*Bufo*) *woodhousii* | MF26121 | Henderson | 0/3 |
| Overlapping anuran | *Craugastor augusti* | MF4226 | Kinney | 0/3 |
| Overlapping anuran | *Craugastor augusti* | MF4398 | Bandera | 0/3 |
| Overlapping anuran | *Eleutherodactylus cystignathoides* | MF36355 | Robertson | 0/3 |
| Overlapping anuran | *Eleutherodactylus cystignathoides* | MF38218 | Bastrop | 0/3 |
| Overlapping anuran | *Eleutherodactylus marnockii* | MF2493 | Bandera | 0/3 |
| Overlapping anuran | *Eleutherodactylus marnockii* | MF6216 | Williamson | 0/3 |
| Overlapping anuran | *Gastrophryne carolinensis* | MF27747 | Bastrop | 0/3 |
| Overlapping anuran | *Gastrophryne carolinensis* | MF27887 | Bastrop | 0/3 |
| Overlapping anuran | *Gastrophryne olivacea* | MF37336 | Hays | 0/3 |
| Overlapping anuran | *Gastrophryne olivacea* | MF4221 | Edwards | 0/3 |
| Overlapping anuran | *Hyla cinerea* | MF36203 | Bastrop | 0/3 |
| Overlapping anuran | *Hyla cinerea* | MF36205 | Bastrop | 0/3 |
| Overlapping anuran | *Hyla versicolor* | MF27743 | Bastrop | 0/3 |
| Overlapping anuran | *Hyla versicolor* | MF27745 | Bastrop | 0/3 |
| Overlapping anuran | *Incilius* (*Bufo*) *nebulifer* | MF37409 | Hays | 0/3 |
| Overlapping anuran | *Incilius* (*Bufo*) *nebulifer* | MF38038 | Williamson | 0/3 |
| Overlapping anuran | *Lithobates* (*Rana*) *berlandieri* | MF35882 | Terrell | 0/3 |
| Overlapping anuran | *Lithobates* (*Rana*) *berlandieri* | MF3713 | Edwards | 0/3 |
| Overlapping anuran | *Lithobates* (*Rana*) *catesbeiana* | MF35335 | Bastrop | 0/3 |
| Overlapping anuran | *Lithobates* (*Rana*) *catesbeiana* | MF35336 | Bastrop | 0/3 |
| Overlapping anuran | *Lithobates* (*Rana*) *sphenocephala* | MF37262 | Robertson | 0/3 |
| Overlapping anuran | *Pseudacris clarkii* | MF30996 | Comal | 0/3 |
| Overlapping anuran | *Pseudacris clarkii* | MF30998 | Comal | 0/3 |
| Overlapping anuran | *Pseudacris streckeri* | MF37410 | Hays | 0/3 |
| Overlapping anuran | *Pseudacris streckeri* | MF37470 | Hays | 0/3 |
| Overlapping anuran | *Scaphiopus couchii* | MF38062 | Ector | 0/3 |
| Overlapping anuran | *Scaphiopus couchii* | MF38231 | Terrell | 0/3 |

**References**

Dixon JR. Amphibians & reptiles of Texas: with keys, taxonomic synopses, bibliography, and distribution maps. 3^rd^ ed. College Station: Texas A&M University Press; 2013.
